# Review of water–energy–food nexus applications in the Global South

**DOI:** 10.1017/wat.2024.8

**Published:** 2024-10-10

**Authors:** Tafadzwanashe Mabhaudhi, Tendai Polite Chibarabada, Cuthbert Taguta, Tinashe Lindel Dirwai, Annah Ndeketeya

**Affiliations:** 1 Centre on Climate Change and Planetary Health, London School of Hygiene and Tropical Medicine, London, UK; 2 Centre for Transformative Agricultural and Food Systems, School of Agricultural, Earth and Environmental Sciences, University of KwaZulu-Natal, P. Bag X01, Pietermaritzburg 3209, South Africa; 3 United Nations University: Institute for Water, Environment and Health (UNU-INWEH), Richmond Hill, Ontario, Canada; 4 Zimbabwe Sugar Association Experiment Station, P. Bag 7006, Chiredzi, Zimbabwe; 5 School of Engineering, University of KwaZulu-Natal, P. Bag X01, Pietermaritzburg 3209, South Africa; 6 International Water Management Institute, Harare, Zimbabwe; 7 Global Water Partnership Southern Africa, 333 Grosvenor Street, Hatfield Gardens, Block A, Pretoria, South Africa

**Keywords:** nexus knowledge generation, environment, climate, resource security, nexus discourse, practice

## Abstract

The study reviewed the applications of the water–energy–food (WEF) nexus for knowledge generation and decision-making in the Global South. The Preferred Reporting Items for Systematic Reviews and Meta-Analyses protocol identified 336 studies from the Web of Science and Scopus datasets. One hundred eighty-five articles applied WEF nexus tools to improve the understanding of WEF nexus interactions and to show the potential of nexus applications. The other articles (151) focused on nexus applications to guide planning and decision support for resource allocation and policy formulation. Environment, climate, ecosystems, land, and socio-economics were other popular nexus dimensions, while waste and economy were considered to a lesser extent. Limitations associated with nexus applications included unavailability of data, uncertainties from data sources, scale mismatch and bias. The inability of nexus tools to capture the complex realities of WEF interactions is hindering adoption, especially for policy formulations and investment planning. Data limitations could be solved using a sound scientific basis to correct uncertainties and substitute unavailable data. Data gaps can be bridged by engaging stakeholders, who can provide local and indigenous knowledge. Despite the limitations, applying nexus tools could be useful in guiding resource management. Limitations associated with nexus applications included – investment planning. Plausible pathways for operationalising the WEF nexus are discussed.

## Introduction

It has been over a decade since the accentuation of the water–energy–food (WEF) nexus at the 2011 Bonn Nexus Conference on the Water, Energy and Food Security Nexus – Solutions for the Green Economy (Hoff, 2011). Driving the WEF nexus is a holistic vision of sustainability that seeks to strike a balance among key strategic resources (water, energy, and food), the different goals, interests, and needs of people and the environment in a world faced with population growth, urbanisation, industrialisation, resource depletion, climate change and degrading ecosystem services (Hoff, 2011). Traditional and sector-based research approaches fall short of addressing the linkages among water, energy, and food resources systems, given that decisions taken in one sector can spill over and affect the other sectors (Bazilian et al., 2011; Lawford, 2019). Nexus approaches facilitate the evaluation of synergies and trade-offs holistically to avoid conflicts, optimise resource allocation, minimise risk on investment and maximise economic returns (Fan, 2016; Mohtar and Lawford, 2016; Mohtar and Daher, 2017; Cai et al., 2018). Since its inception, the WEF nexus approach sparked interest among the academic and development communities, resulting in policy dialogues and the development of a wide range of frameworks and tools for analysing the WEF nexus to guide decision-making for improved governance across sectors (Klümper and Theesfeld, 2017; Ramaswami et al., 2017; McGrane et al., 2019; Simpson and Jewitt, 2019).

Significant progress has been made in developing WEF nexus tools for different spatial and temporal scales, contexts and users. The abilities, strengths, and shortcomings of current techniques in capturing the nexus approach and its different components have been the subject of several reviews (Kaddoura and el Khatib, 2017; Dai et al., 2018; Shannak et al., 2018; McGrane et al., 2019; Endo et al., 2020; Purwanto et al., 2021). Some of the weaknesses identified with the WEF nexus was the omission of other important sectors that influence resource security, such as land, ecosystems and climate change (Zhang et al., 2018; Dalla Fontana and Boas, 2019; Bian and Liu, 2021). Taguta et al. (2022) reported the lack of basic and requisite characteristics in documented WEF nexus tools, including ready availability, geospatial analytic capabilities, and applicability across different scales and locations. The lack of data that supports efforts to understand system boundaries and spatial dimensions was also cited as a barrier to the application of the WEF nexus approach (McCarl et al., 2017a; 2017b; Gomo et al., 2018; Lawford, 2019). Additionally, WEF nexus methodologies often fail to reflect the study region’s uniqueness and to incorporate appropriate activities among different contexts (Zhang et al., 2018; Dalla Fontana and Boas, 2019; Bian and Liu, 2021). For example, the Global South and Global North have differing development trajectories, thus unique priorities and activities pursuing water, energy and food resource security (Reidpath and Allotey, 2019; Kowalski, 2020).

As the nexus approach grows and expands, there has been rising interest to shift from theory to practice, thus applying the nexus for implementing technical solutions, resource management and policy development (Liu et al., 2017; Purwanto et al., 2021). Previous studies have discussed WEF nexus tools (frameworks, discourses and models) comprehensively without linking them to applications (Kaddoura and el Khatib, 2017; Dai et al., 2018; Shannak et al., 2018; McGrane et al., 2019; Endo et al., 2020; Purwanto et al., 2021). This study focuses on WEF nexus applications within the Global South as the region is associated with high levels of poverty, high population growth rates and a high prevalence of food insecurity, among other issues (Akinbode et al., 2022; Fuseini et al., 2024). The specific objectives are to (i) identify how WEF nexus tools have been applied to facilitate knowledge generation and decision-making in the Global South, (ii) identify nexus nodes (units of the nexus structure) being considered under different contexts in the Global South, (iii) identify limitations in the application of WEF nexus tools for decision making and knowledge generation in the Global South and iv) propose pathways for operationalising the WEF nexus that are contextualised for the Southern African Development Community (SADC) region.

The paper is structured as follows: after this introduction, ‘Materials and methods’ section describes the method and materials used, including data sources, data curation, and analysis of the data. ‘Results and discussion’ section presents the results: (i) a synopsis of the database, (ii) how WEF nexus tools have been applied for knowledge generation and decision support, and (iii) challenges associated with WEF nexus applications in the Global South. ‘Way forward and recommendations: Pathways towards operationalising the WEF nexus in Southern Africa’ section provides plausible pathways for operationalising the WEF nexus. ‘Study limitations’ section highlights the study’s limitations, and ‘Conclusions’ section is the study’s conclusion.

## Materials and methods

### Definition of terms

In this study, application refers to the published use of the WEF nexus (concept, discourse, model, etc.) in assessing real-life circumstances or status quo assessment or simulating and modelling hypothetical scenarios (Saundry and Ruddell, 2020). The WEF nexus can serve multiple roles, such as a conceptual framework, an analytical tool, or a discourse (Keskinen et al., 2016). Firstly, the WEF nexus conceptual framework leverages an understanding of WEF linkages to promote coherence in policy-making and enhance sustainability. Secondly, WEF nexus analytics systematically use quantitative tools (e.g. quantitative models) and/or qualitative methods (e.g. participatory stakeholder workshops) to highlight and understand interactions among water, energy, and food systems. Thirdly, the nexus discourse can facilitate problem-framing and promote cross-sectoral collaboration (Keskinen et al., 2016; Albrecht et al., 2018).

One of the study objectives was to identify the extent to which the WEF nexus was used to generate knowledge and make decisions. According to the Oxford Dictionary, knowledge is “facts and skills acquired through experience or learning; the theoretical or practical understanding of a subject matter”. For context, this study applied the definition to assess and map the application of WEF nexus tools, frameworks and discourse to generate facts and tools that inform the better management of WEF nexus resources. This study targeted the whole knowledge generation value chain, i.e., knowledge generation as a process, output, and outcome in the WEF nexus theatre of activity (Mitchell and Boyle, 2010). Each value chain component is defined in Table 1.

The study also defined decision-making as situations whereby stakeholders are individually or collectively required to make choices based on the available facts or information (Hill and McShane, 2008). The decision-making process can be a bottom-up or top-down approach.

### Search strategy

The review was guided by the Preferred Reporting Items for Systematic Reviews and Meta-Analyses (PRISMA) protocol (Moher et al., 2009; Page et al., 2021) (SF1). The Population, Intervention, Comparison and Outcomes (PICO) framework was used to develop literature search strategies to ensure comprehensive and bias-free searches (Table 2).

A literature search was conducted in two databases [Scopus and Web of Science Core Collection (WoS)] (The last search was on 02 April 2024). The search criteria in the two databases (Scopus and WoS) are presented in Table 3. In the WoS platform, we searched all editions of the WoS core collection.

Literature from the Global South was screened during abstract screening. The classification of studies between Global North and Global South was based on Dados and Connell (2012), the United Nations Conference on Trade and Development (2018) and Kowalski (2020). The search identified 1,451 and 921 articles from Scopus and WOS, respectively. Together, the initial database comprised *N* = 2,372 articles. A duplicate check in MS Excel identified 703 duplicates that were immediately removed. Consequently, 1,699 articles were screened by title and abstract (Supplementary Figure [SF] 1). Consideration was given to peer-reviewed papers (articles), scientific book chapters, papers and proceedings written and published in English. The date of publication was limited to 2011 (birth of WEF nexus) to the date of the last search (02 April 2024), while the geographic scope, journal disciplines and impact factors were kept open to capture all WEF nexus case studies.

### Screening and bias reporting

Three authors (T.P.C., C.T. and T.L.D.) were assigned to screen the abstracts independently. The screening was done by scoring an article’s relevance against a five-point Likert scale (1 – extremely irrelevant and 5 – denoting very relevant). The Koutsos et al. (2019) criteria for ranking article relevance was modified to develop scoring criteria for the articles and facilitate screening (Table 4). Articles that were scored 3 and above by all authors were automatically included. Articles scored 3 or above by at least two authors were also automatically included. Where only one author scored 3 or above, it was resolved by discussion. Articles that were scored 2 and below by all authors were excluded. Articles reporting WEF nexus applications from the Global North were scored 1 as they were extremely irrelevant for this review. Secondary articles, such as reviews, were also scored 1 as they summarised existing studies, and this study delved into primary research (Table 4). The screening favoured publications capturing any nexus and applying WEF nexus concepts, discourse and tools in addressing real-life situations from the Global South. Of the 1,699 articles, 815 were from the Global North, while 127 were reviews and other secondary articles. The remaining 757 articles included 336 studies that applied the WEF nexus approach for any reason (to gain insights, solve a problem, plan, identify factors and aid in decision-making.). These 336 studies were subjected to data extraction by one author (T.P.C.).

### Data collection

A data extraction sheet was designed in MS Excel. Key data on the selected papers were extracted from the eligible studies and organised in the data extraction sheet. The data items were organised in columns, including publication details (author, year, title), objective, case study (location, country, continent, region), scale (spatial, temporal), nexus nodes, involvement of stakeholders and analytical or modelling tool used. The World Bank regional units (Africa, South Asia, Middle East, Latin America and the Caribbean, Central Asia, and East Asia) were used to categorise regions. The spatial scales were classified as household, field, farm, community, village, town, city, municipality, district, metropolitan, provincial, national, catchment, watershed, river basin, aquifer, continent, and global. Where studies explicitly highlighted the limitations of the application, this was captured.

### Data items and analysis of studies

To facilitate data visualisation and trend analysis, Bibliometrix and Biblioshiny packages from the R language environment were used to map research hotspots and to develop an international collaboration network map. The temporal two-dimensional multi-correspondence analysis (MCA) plot was used to visualise the WEF nexus case studies approach from 2011–2024. A trend analysis was done based on abstracts and keywords. The word tree was prepared using Jason Davies’ Word Tree (Wattenberg and Viégas, 2008).

## Results and discussion

### Overview of WEF nexus application studies in the Global South

**Figure 1:**
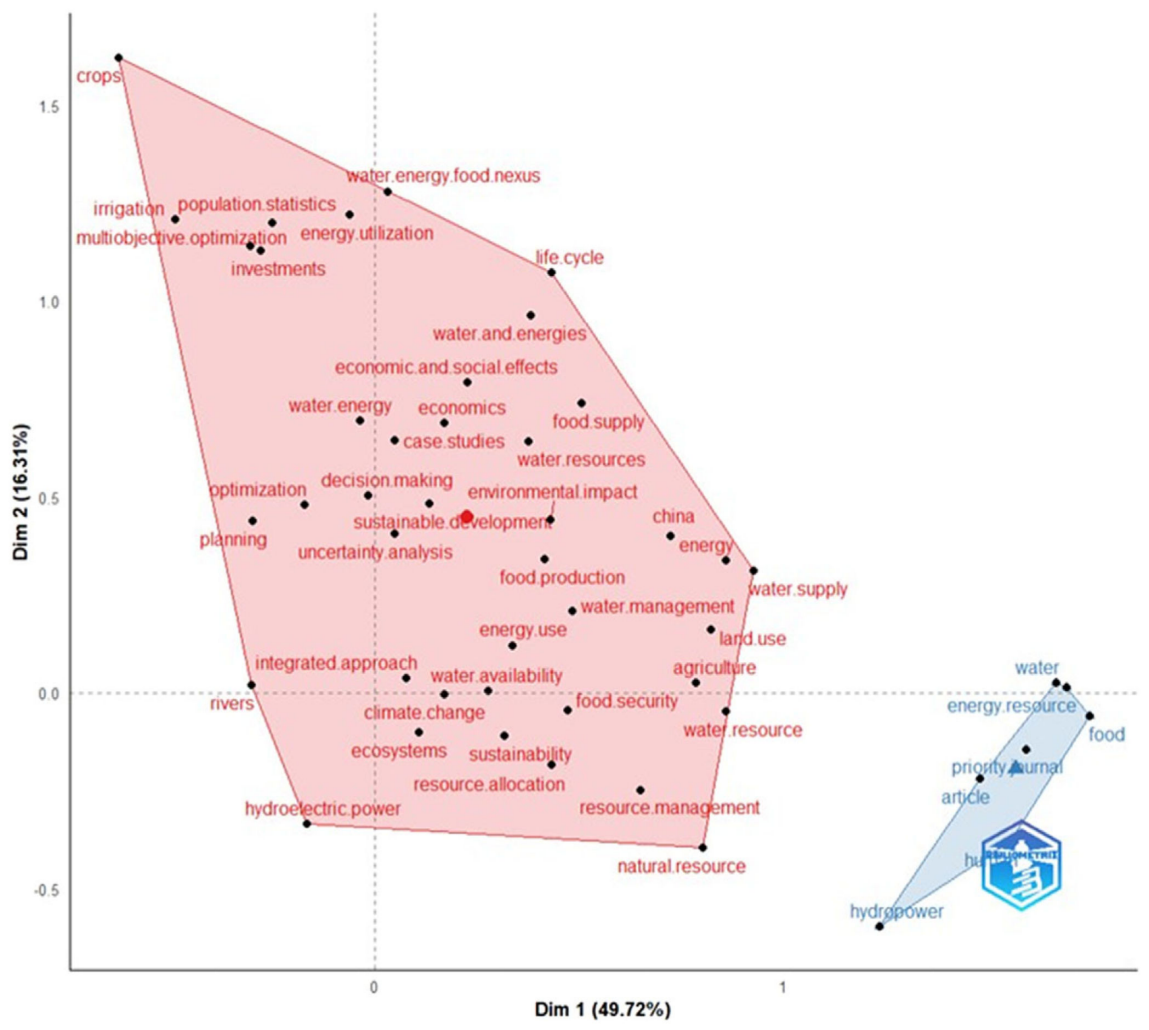
Temporal two-dimensional visual showing the red and blue cluster grouping words according to WEF nexus associations with case studies that applied the WEF nexus approach to create knowledge or for decision support. The red cluster (*n* = 39 words) had higher word association than the blue cluster (*n* = 6 words).

The conceptual structure map showed that the best size reduction between the two dimensions accounted for 66% of the total variability, i.e., 49.72% and 16.31% for dimensions 1 and 2, respectively (Figure 1). The conceptual structure map showed two distinct clusters (red and blue), and in the plot, the closer the points are to each other, the more similar subject matters they cover in their respective sectors. For example, the sectors Dim 2 (0.0–1.5) and Dim 1 (origin-0) with n= 12 words show a close relationship amongst the words water–energy, irrigation, crops, investments, optimization and energy utilisation, to mention a few. In this context, we observed that, to a greater degree, several cases were linked to economics, economic and social effects, water resources and food supply (Figure 1). China is the only country that appeared in the keyword mapping (Figures 1 and 3), implying that the database comprises many studies conducted in China compared to other countries. An earlier review (Correa-Porcel et al., 2021) and a recent one (Rhouma et al., 2024) reported that China ranks second to the USA regarding the total number of WEF nexus articles published in each country. This could be because China’s GDP has been increasing by 9% annually, making it the fastest-growing economy in the Global South (https://www.worldbank.org/en/country/china/overview).

**Figure 2:**
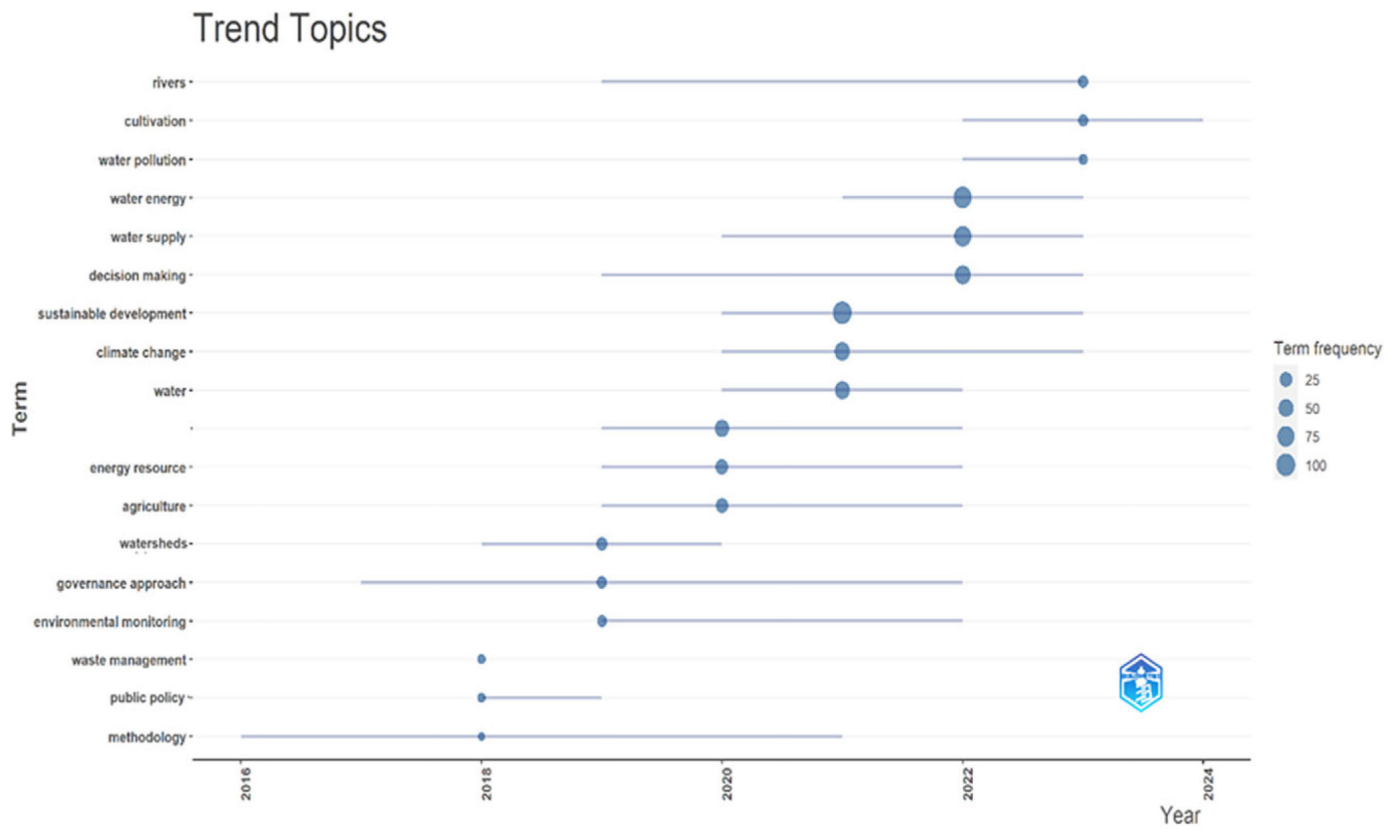
Trend topics associated with the WEF Nexus applications database. The trend diagram depicts the evolution of different subject matters related to the WEF nexus research frontier. After the year 2022, decision-making dominated the WEF nexus space. Decision-making is part of the knowledge generation value chain, i.e. process, outcome and output.

Trend analysis gave an insight into trending topics based on word occurrence (Figure 2). Between 2020 and 2022, sustainable development and the WEF nexus resources dominated the discourse. This could mean that the WEF nexus is integral in the quest for integrative sustainable management of resources and economic development. A key WEF nexus challenge is to develop policies that support the sustainability of water, energy, and food resources while ensuring universal access to these resources (Simpson and Jewitt, 2019). Post 2022, the relatively dominant words included rivers, cultivation, and water pollution were dominant; this is potentially attributed to river basins being good examples where water, energy, and food interconnect as they supply freshwater, regulate water flow and quality, and generate energy (such as hydropower) (Ringler et al., 2018). The word methodology was prominent post-2016–2016, which we assume could be attributed to the development of WEF nexus tools. The evolution of the WEF nexus as an integrative approach gained traction post-2016. After 2018, the term decision making became more prominent.

### Application of nexus approaches

From the databases, we categorised the overall purpose of nexus applications. Two major themes were used: (i) to improve understanding and generate knowledge on WEF interactions and (ii) as a decision support tool.

### Understanding and knowledge generation of WEF nexus interactions

Studies under this category aimed to generate knowledge on nexus interactions by quantifying WEF indices at varying scales and understanding the impact of resource allocation at different scales. This knowledge generation approach was mainly focused on the outcomes and outputs components of the knowledge generation value chain. These studies are important to facilitate adopting the approach through evidence on quantitative and qualitative relationships among the sectors and highlighting the advantages of nexus vs silo approaches (Naidoo et al., 2021). Most of the studies in the database (*N* = 185) were under this category, which could be explained by the fact that the nexus research is shifting from theory to practice. Most studies have been focused on testing and validating the ability of nexus tools to capture intersectoral linkages, thus offering practical recommendations for their application as decision-support tools or to address specific challenges (Supplementary Table [ST] 1).

Taghdisian et al. (2022) explored the potential of the ‘Multi-Scale Integrated Analysis of Societal and Ecosystem Metabolism’ (MuSIASEM) framework for resource analysis at the river basin scale. Their results showed that the framework would fill an important gap to guide nexus governance in the region; however, there was a need to co-produce analysis with social actors, and there was a need for good-quality basin data. Stein et al. (2018) analysed how actors involved in the governance of WEF are embedded in social networks. They highlighted that actors are not simply disconnected, but there are hierarchical structures that result in coordination challenges despite visible theoretical cross-sectorial linkages. By combining different methods (gridded water balance model and GIS), Daccache et al. (2014) quantified irrigated regions’ water demand and energy footprint. The study demonstrated the possibility of combining different methods to integratively analyse the WEF nexus and facilitate an understanding of the water, energy, and food nexus that could be used for policy formulation on irrigated agriculture (Daccache et al., 2014). Another knowledge generation as an output scenario was done by Taguta et al. (2023). The authors (Taguta et al., 2023) developed a geospatial integrative iWEF 1.0 model to assess WEF nexus usage across multiple scales for building resilience and adaptation strategies.

### Planning and decision support

**Figure 3:**
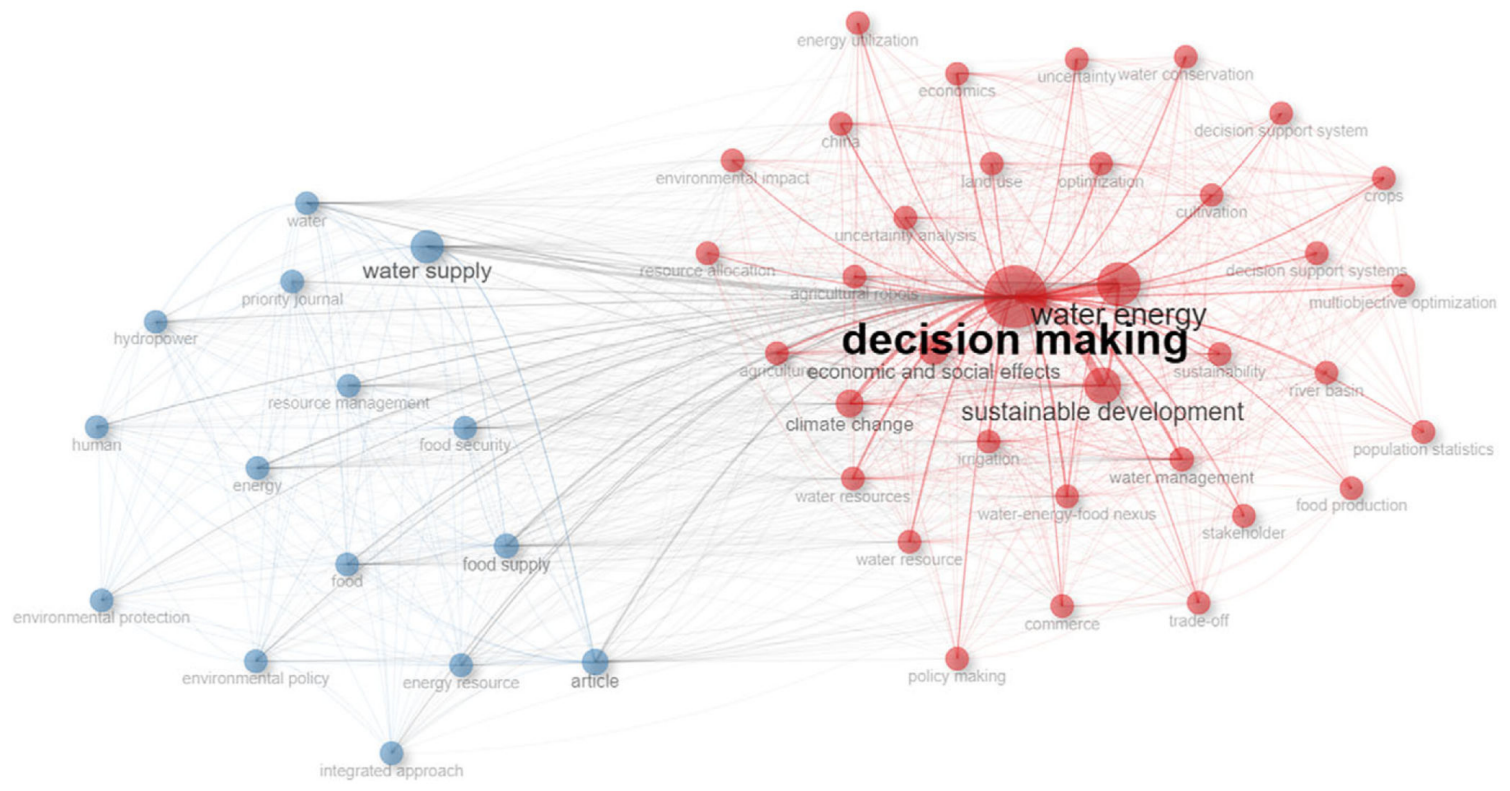
Decision-making linkages co-occurrence network. The main red cluster had decision-making at the centre and was effectively and directly linked to 32 socio-economic, socio-political-ecological related words. The minor blue cluster centred on water supply was linked with decision-making for food supply, hydropower, and environmental protection, to mention a few.

Planning is concerned with setting objectives and targets and formulating plans to accomplish the objectives. It involves logical thinking and rational decision-making. Nexus tools have been applied to evaluate options and scenarios for the identification of optimal decisions for resource allocation at different scales and contexts. The modified search strategy incorporating WEF nexus and decision-making produced a co-occurrence network in which decision-making was strongly linked to water supply, economic and social effects, multiple objective optimisation, population, policy making, and energy utilisation, to mention a few (Figure 3).

In addition to optimal resource allocation, nexus approaches have also been valuable in identifying the most economical strategies, such as energy utilisation, water management, water conservation, and resource allocation, to mention a few (Seeliger et al., 2018; Das et al., 2020; Siderius et al., 2022). The authors hypothesise that the application was based on the ability of the WEF nexus to identify trade-offs and synergies that facilitate decision-making at the operational and policy levels. An example of a nexus approach for planning purposes was when the future allocation of land and water resources for agriculture and hydropower generation in the transboundary upper Blue Nile (UBN) basin was determined using a WEF nexus framework by Allam and Eltahir (2019). Li et al. (2021a) applied the WEF nexus approach at the field scale to identify a sustainable cropping system to maximise crop production while reducing energy consumption and water depletion. Also, under agricultural development, Guo et al. (2022) applied a nexus approach to determine sustainable agricultural irrigation development at the river basin scale without negatively affecting hydropower generation and other water uses. In another study, a nexus tool was used to identify stakeholders that would participate in a programme to rehabilitate a reservoir (Melloni et al., 2020). In the context of decision support, the nexus that included climate as an important node was applied to identify adaptation strategies and to ensure the resilience of current WEF policies to climate change (Mabhaudhi et al., 2019; Qi et al., 2021; Yue et al., 2021a).

### Nexus nodes

**Figure 4:**
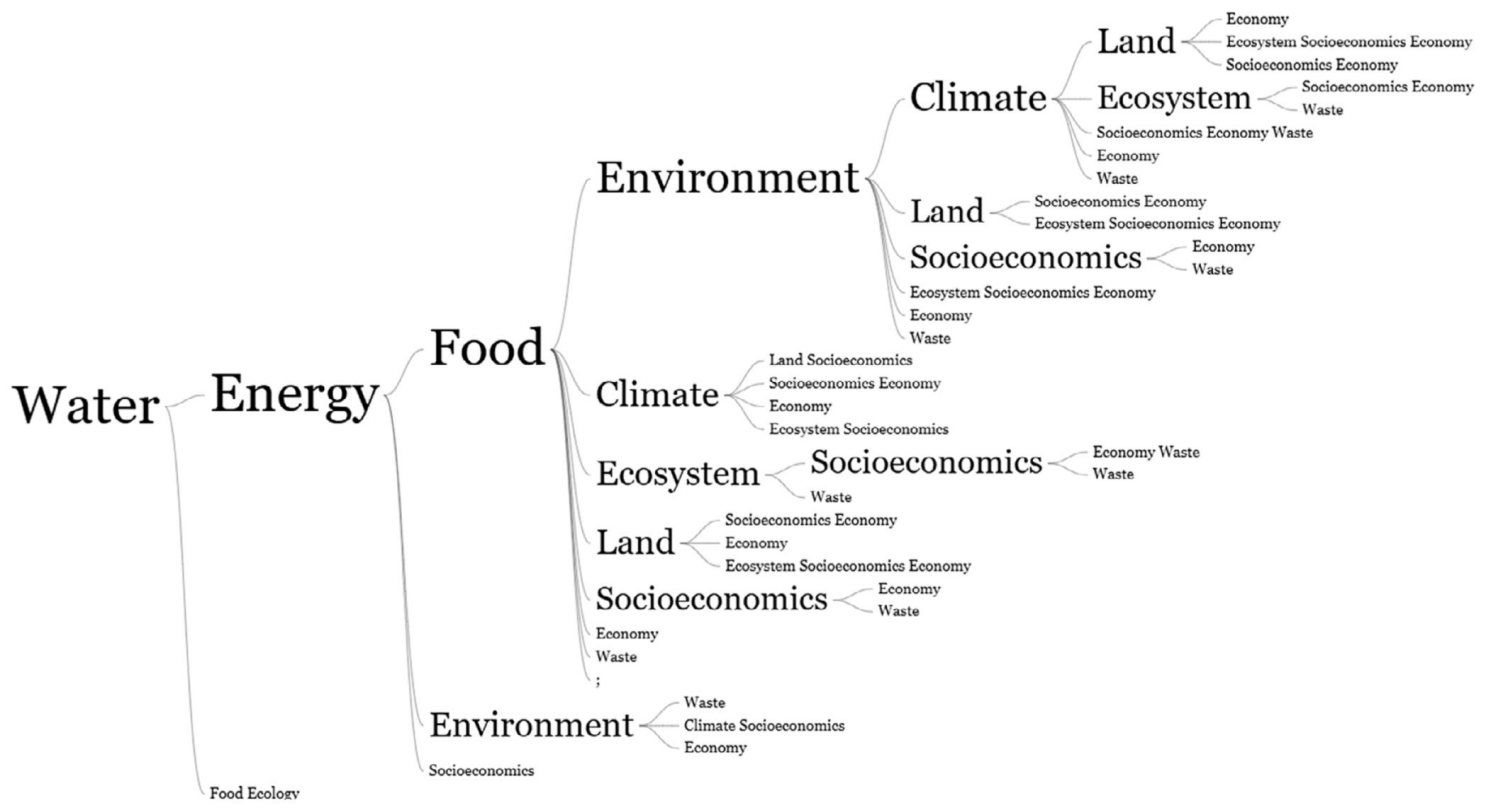
Word tree of nexus nodes considered in nexus application studies in the Global South.

Building from the World Economic Forum in 2011, the nexus was recognised from the water–energy–food nexus perspective. While this has been the most common definition, there have been varying interpretations in different sectors and contexts. Nexus thinking is an analytical approach that seeks to identify and quantify the links between the nexus nodes. In this review, nexus concepts analysed in each study were captured and subjected to a word tree to visualise other nexus nodes that have been used. Socio-economics was grouped to represent issues concerning human livelihoods, health, culture and general well-being. Results show that WEF environment has been quite popular in nexus studies (Figure 4). Environment has been a popular node as it addresses broader issues to do with land use, greenhouse gas emissions, carbon footprint and biodiversity (Dhaubanjar et al., 2017; Nie et al., 2019; Lahmouri et al., 2019; Melloni et al., 2020; Malagó et al., 2021; Zhang et al., 2021; Correa-Cano et al., 2022; Taghdisian et al., 2022). According to Simpson and Jewitt (2019), the environment is an irreplaceable foundation for the WEF nexus as it underpins the security of WEF resources. Figure 4 shows that after the environment, there were many other branches (climate, land, socioeconomics) and sub-branches where climate was linked to land and ecosystems.

Water–energy–food–climate, water–energy–food-ecosystems, water–energy–food–land and water–energy–food-socioeconomics were popular nexus definitions (Figure 4). It was observed that water–energy–food–climate was used in studies focussing on climate change adaptation and resilience of households/communities to climate change (Adom et al., 2022; de Souza and Versieux, 2021; Diriöz, 2021; Lee et al., 2020; Pardoe et al., 2018; Rasul and Sharma, 2016; Yang, et al., 2018; Yue et al., 2021a). Like the environment, the ecosystem broadly refers to issues of biodiversity, ecology and the sustainability of the environment (AbdelHady et al., 2017; Karabulut et al., 2019; Müller-Mahn and Gebreyes, 2019; Muthee et al., 2021). Studies addressing the water–energy–food–socioeconomics nexus were more popular at river basin and transboundary scales where livelihoods are directly impacted, especially for small-scale agriculture, fishing and tourism. For example, the consideration of health in the WEF nexus during dam development was highlighted following the transmission of *Schistosoma spp*. parasites in humans in the Senegal River Basin (Lund et al., 2022).

Waste, both urban and economic, was not often used relative to the environment, ecosystem, climate, and socioeconomics (Figure 4). We observed waste to be more popular in studies on urban development. This was also similar to the economy. These nodes were more popular in China, where the WEF nexus was more popular in the context of urban planning and urban metabolism (Li et al., 2016; Niva et al., 2020; Xu et al., 2020; An et al., 2021; Ma et al., 2021; Qi et al., 2022; Zan et al., 2022). In some water–energy–food–waste studies, wastewater was proposed for irrigation purposes, thus promoting a circular economy characterised by recycling and reducing pressure on freshwater resources (Lahlou et al., 2020; Das and Chirisa, 2021; Ramirez et al., 2021).

### Limitations of WEF Nexus applications in the Global South

Data availability allows stakeholders to take stock of economic and environmental resource availability, use and management (Naidoo et al., 2021). Various studies highlighted data limitations as one of the major challenges in the real-life application of nexus approaches. This could have been a result of the unavailability of the data (Perrone and Hornberger, 2016; Ozturk, 2017; Bellezoni et al., 2018; Gaddam and Sampath, 2022; Li et al., 2022), uncertainties stemming from data sources (Perrone and Hornberger, 2016; Ozturk, 2017; Li et al., 2021a), scale mismatch (Feng et al., 2022; Han et al., 2020; Nie et al., 2019) and biases especially in the case of qualitative data (Namany et al., 2021). Naidoo et al. (2021) emphasised data scarcity at sub-national scales and highlighted other data challenges at all scales related to heterogeneity, disparity, plurality, varied data collection and storage methods, and different data quality and standards. Some governments and organisations in the Global South guard WEF-related data as a matter of national security and sovereignty, while some charge thousands of dollars for long-term data, for example, 30-year multi-station daily climate data. Some custodians who commercialise such data as climate and hydrological claim that selling such data is their only source of income for meeting operation costs towards sustainable data collection without funding from the government and external sources. In a study to track the urban energy-water-land flows within local, regional, national, and global supply chains from the production and consumption perspectives. The sectoral data provided in the World input-output table (Timmer et al., 2015) were highly aggregated and limited the reliability of the results (Meng et al., 2022). When national statistics were used, they misrepresented regional and local variations for various indicators of food, energy, and water security (Mohammadpour et al., 2019). In addition, analyses of nexus using political boundaries and administrative-area levels (provinces, metropolitans) limit the ability to extend to other nodes (environmental and ecosystem) that transcend political boundaries.

Concerning qualitative data, Namany et al. (2021) reported that experts’ judgements can influence the estimation of importance scores. In addition, when experts from a single sector conduct scoring, they are subjective and not in the interest of nexus considerations (Namany et al., 2021). The strength of any quantification tool for nexus depends on the strength of the data. Where data is limited, or there are uncertainties, all the assumptions applied to cover the lack of data and to correct any uncertainties should be performed through sound scientific fundamentals (Bellezoni et al., 2018; Sun et al., 2022).

Another limitation in applying the nexus approach to real-life case studies was the inability to capture the complexities of those interactions in reality and entirety (Perrone and Hornberger, 2016; Bakhshianlamouki et al., 2020; Shi et al., 2021; Wang et al., 2021). A quantification of WEF nexus interactions in the Brahmaputra River Basin, South Asia, using a hydro-economic water system model showed the potential to provide advanced knowledge to inform policy dimensions of natural and social driver changes impact on the WEF nexus (Yang et al., 2016a). However, the basin’s reality was more complex than captured by the model, which could cause policymakers to resist adopting such an analysis. The model did not consider capital and operational costs of water diversions, the loss of other ecosystem services and the diurnal variations in streamflow (Yang et al., 2016a). To better represent the real world, agent-based water resources system models are more accurate than centralised optimization frameworks. However, agent-based water resources system models require comprehensive datasets with inherent weaknesses and limitations (Yang et al., 2016a).

## Way forward and recommendations: pathways towards operationalising the WEF nexus in Southern Africa

While the literature search focused on the Global South, we propose pathways for operationalising the WEF nexus contextualised for the SADC region. The SADC Regional Strategic Action Plan on Integrated Water Resources Development and Management Phase V highlights the importance of the WEF nexus and the need to have integrated planning and implementation at both a regional and national level (SADC, 2023) Due to the global approach applied in the study, the pathways are generalisable to many global South regions that share a similar context as the SADC. Plausible pathways towards operationalising the WEF nexus are summarised using the Theory of Change (ToC) framework developed by Naidoo et al. (2021) (Table 5). The crux of the framework was to develop a platform for cross-sectoral dialogues and institutions that can guide key stakeholders to identify and prioritise solutions together from an overall nexus perspective (Naidoo et al., 2021). A ToC clarifies the connections between a given intervention and its outcomes, thus creating a better understanding of what is being implemented and why (Table 5).

## Bridging the science-policy-practice gap

The inherently vulnerable Global South continues to suffer from thirst, darkness and hunger despite the promises of WEF security through historical sectoral and integrated approaches (Ringler et al., 2013). The security of WEF resources challenges the region and struggles to achieve SDGs, and this is intensified by historical inequities, injustices and imbalances in access and distribution (Murombedzi, 2016). For example, Chile currently leads the Global South countries in the overall progress towards achieving all 17 SDGs, but it has a score of 78.22% and is ranked 30^th^ out of all 193 UN Member States (UN, 2022). South Africa demonstrates how historical inequalities contribute to distribution and access to WEF resources and SDG attainment. South Africa is ranked the world’s most unequal country, ranking first out of 164 countries (International Center for Transitional Justice, 2022). This has consequently led to the country being ranked 110^th^ (SDG index score = 64%, global average = 66.7%) with stagnancy in SDGs 2 (zero hunger) and 7 (affordable and clean energy) and moderate improvements in SDG 6 (clean water and sanitation) (UN, 2022). Disparities exist in access to WEF resources between South Africa’s urban and vulnerable peri-urban, rural, and informal settlements (StatsSA, 2019; 2023). Zimbabwe is ranked 138^th^ (SDG index score = 55.6) with stagnancy in SDG 2 (zero hunger), a decrease in SDG 6 (clean water and sanitation) and moderate improvements in SDG 7 (affordable and clean energy). Implementing the WEF nexus approach with equal consideration between social groups can redress inequity and inequality through just transition, social justice, and sustainable and equitable development of society towards low-carbon economies in the Global South (Murombedzi, 2021). Nexus implementation must consider outcomes for the poor and vulnerable to not compromise their well-being (Ringler et al., 2013).

Global changes in climate exacerbate the WEF challenges in the Global South, which are also amplified by pandemics (e.g., COVID-19) and conflicts (e.g., Russia-Ukraine) that disrupt the supply chains of food (grains, cooking oil), fertiliser and energy (fuel, gas). Interlinkages between climate change and WEF resources are forward and backwards because climate change affects WEF resources and sectors (Rasul and Sharma, 2016). In the current era of climate change, global warming, and climate variability, the need arises to consider the climatic dimension in the nexus thinking equally for an inter-sectoral response. Similarly, climate mitigation and adaptation should be planned and implemented from an integrated nexus perspective to minimise maladaptation. Thus, a paradigm shift from a vicious cycle of conventional sectoral management approaches to a potentially virtuous cycle of implementing nexus approaches is more likely to accelerate the inclusive achievement of the COP21 Paris climate change commitments and SDGs. An equally important dimension in the nexus is the environment. Nexus deliberations must account for environmental outcomes to preserve and maintain ecosystems that underpin the security of WEF resources (Ringler et al., 2013).

The WEF nexus promises to simultaneously and collectively achieve the security of water, energy and food through improved allocation and efficiencies. The approach has progressed significantly, if not rapidly, in the research and policy space, although implementation is still in its infancy. Science through the research dimension has enhanced understanding and knowledge of the concept, developed relatively abundant tools, and amassed volumes of evidence. Similarly, the policy space has congregated decision-makers in dialogues that promote collaboration, sharing and integration. The Global South must contextualise the WEF nexus tools, evidence and relevant policies into actionable strategies, programs and actions that can be implemented, from pilots to full-scale, for just and inclusive transitions and transformations that leave no one behind in sustainable development. For example, dialogue findings can be used to develop coherent nexus-friendly policies. In contrast, lessons from nexus and scenario planning studies can be used to develop harmonised and integrative incremental and transformative pathways whose scenarios can be exploratively simulated by WEF nexus modelling tools. Promisingly optimal intervention(s) that optimise synergies and minimise trade-offs are combined into WEF investment packages, which are piloted so that their real-life impacts can be evaluated and monitored. Challenges are noted, lessons are learnt, and improvements are made for deep-, out- and up-scaling.

### Addressing data needs to enable nexus applications

There is a high demand for quantitative and qualitative data and information to apply nexus models and frameworks. Nexus applications have relied heavily on secondary databases, which are largely sectorial and limited in depth, accuracy and spatial and temporal scale. The type of data, its format, and its accuracy are important for developing nexus tools and their usefulness and reliability in solving real-life challenges. Researchers need to clearly outline data needs at different scales from relevant authorities to satisfy WEF tools. This should be complemented by standardised data collection protocols that can guide data collection and ensure good quality and uniform data comparable across scales, space and time is obtained. Government departments, academia, research organisations, and development agencies are encouraged to collaboratively follow strict data curation practices to ensure that high-standard data is available and easily accessible. The development of nexus tools should also balance both simplicity and accuracy with minimal data input requirements.

### Building and strengthening capacity for WEF nexus adoption and application

WEF nexus research still largely exists at an academic and scientific level, especially in the Global South (Lazaro et al., 2022). The move to Open Access is changing this. However, much research is still pay-walled, and some targeted end users of the research findings, such as policymakers, lack the skills to understand and translate scientific evidence (Cairney and Oliver, 2017; Gollust et al., 2017). From the perspective of some policymakers, some main barriers to accessing scientific literature include time to read papers and difficulty in understanding technical language (Karam-Gemael et al., 2018). Thus, concise packaging of the relevant information is needed, paying particular attention to what information needs to be transferred to policymakers and how to package and present it to improve the likelihood of using it (Strydom et al., 2010). There is a need to build and strengthen the capacity of researchers, practitioners and policymakers to jointly undertake nexus assessments and translate the evidence into policy and practice outcomes, especially in the context of investment and sustainable development planning. Such capacity-building should consider the regional context and integrate the biophysical and socio-ecological systems to enhance people and planetary benefits across multiple scales (from farm or village to country and region). This requires transdisciplinary approaches that cut across disciplines, sectors and actors to ensure impact.

## Study limitations

The review used the PRISMA guidelines to identify, select, appraise, and synthesise studies. Due to the use of predefined search terms and inclusion criteria, some literature may have been excluded. The search was also done in scientific databases (WoS and Science Direct), thus excluding other potential sources of ‘grey literature’ such as reports and theses that are not all included in scientific databases. During synthesis, the study identified two major drivers of nexus applications: (i) to improve understanding and to generate knowledge on WEF interactions; and (ii) for planning purposes and as a decision support tool. While the categorisation was subjective, the authors represent expertise in the science and public space and are experts on the WEF nexus. While the pathways are contextualised for southern Africa, the literature review was at a Global South level due to limited research outputs specific to the region.

## Conclusions

We reviewed WEF nexus applications in the Global South to develop pathways for WEF nexus operationalising at a regional scale. There is a drive to shift from nexus theory to practice. Hence, there has been a surge in studies aiming to validate nexus tools and improve understanding of nexus interactions at different scales (Al-Saidi et al., 2023). These studies have been valuable for knowledge generation and provide optimism on the possibilities of nexus approaches for solving real-world problems.

Nexus nodes are not limited to the default water, energy and food. The approach has extended to address global challenges such as climate change, environmental degradation, land scarcity, and livelihoods. This highlights the catalytic nature of the WEF nexus approach and how it can facilitate broader systemic change.

Data availability, quality, and scale mismatch concerns could hinder applying nexus approaches to solve problems. However, this should not be a deterrent. Data limitations can be overcome through sound methods when making assumptions and statistical methods to (dis)aggregate and down/upscale data to suit a specific scale.

The inability of nexus approaches to capture reality was also cited as a major limitation; however, we believe no model is perfect and is a true representation of reality. Models should aim to capture important aspects of the system and accurately respond to changes in input variables while addressing the questions and objectives in focus. That ability to respond to input variables and show trends is important for planning and decision-making. Recommendations towards operationalising the WEF nexus include bridging the science-policy-practice gap, generating data for developing and applying nexus tools and building capacity within students, researchers and practitioners. While these recommendations are contextualised for southern Africa, they are transposable to other global South regions with a similar development context.

## Supplementary Material

The supplementary material for this article can be found at http://doi.org/10.1017/wat.2024.8.

supplementary material 1

supplementary material 2

## Figures and Tables

**Table 1:** Knowledge generation value chain, i.e. process, output and outcome

Component	Definition	Contextualised examples
Process	“the method through which new ideas are generated, incorporating activities, interactions and other organisational mechanisms” (Styhre et al., 2002; Mitchell and Boyle, 2010).	Activities and steps undertaken in the pursuit of new knowledge. Activities include stakeholder meetings to identify other dimensions influenced by WEF nexus resources utility, e.g., WEF–Health–Biodiversity.
Output	“the generation of enriched new ideas, manifest as, for example, a description, graphic or verbal depiction”(Johnson, 2002; Parent and Gallupe, 2000 as cited by Mitchell and Boyle, 2010)	This includes immediate knowledge of products and idea presentations. For example, the novel integrative geospatial iWEF tool for supporting decision–making on WEF nexus resources utility for building resilience (Taguta et al., 2023).
Outcome	“the generation of an object, which is demonstrable, such as a routine, prototype or publication, and which represents the realisation of a new idea” (McFadyen and Cannella, 2004; Nonaka, 1994 as cited by Mitchell and Boyle, 2010).	This involves adding value to outputs that facilitate change in methodologies, routines, and product prototypes (Malhotra and Majchrzak, 2004). For example, upgrading the iWEF tool to incorporate more indicators and upgrade decision–making to other multi–criteria decision–making techniques besides the AHP.

**Table 2:** PICO strategy used to develop the search strategy

PICO	Description
Population	WEF nexus
Indicator	WEF nexus real–life applications and WEF nexus case studies
Comparison	N/A
Outcome	WEF nexus applications and case studies

**Table 3:** Terms used in searching literature in Scopus and WoS databases

Search topic (first row)	Search topic (second row)	Search topic (third row)
(water–energy–food)	AND nexus	(case study* and application*)

**Table 4:** Manuscript scoring based on the study’s relevance (modified from Koutsos et al., 2019)

Relevance	Type research	Study design	Study type	Evidence
Very Relevant (5)	Applied research, Adaptive research	Case studies, model simulations, field trials	Experimental/observational	Substantiated
Relevant (4)	Applied research,	Experiments, field trials, model simulations	Experimental/observational	Substantiated
Moderate (3)	Applied simulation	Case studies, simulations	Observational	Partially substantiated
Irrelevant (2)	Opinion pieces	Qualitative research, opinion papers, reports of expert committees	Descriptive	Unsubstantiated qualitative analysis and opinions
Very irrelevant (1)	Reviews		Narrative	Unsubstantiated qualitative analysis and opinions

**Table 5:** Accelerating WEF Nexus transition from theory to practice in the SADC region

WEF Nexus Operationalisation Framework	Challenge	Research gap/needed	Integrated approach	Desired outcome
Knowledge and Innovation	Lack of integrative nexus analytical tools (limited to individual sector(s), Missing feedback, Lack, mismatch and heterogeneity of data and information	Improvement and integration of existing tools, development of integrative tools	Integrative nexus analytical tools, multi–model frameworks, multidisciplinary and trans–disciplinary approaches, citizen–generated data	WEF nexus metrics and indices Integrative nexus analytical tools Co–generated data
Applications	Lack of evidence on success and pitfalls	Case studies and demonstration pilots Science–based evidence	Testing, monitoring and evaluation from a nexus perspective Scenario planning, modelling and simulation	Guidelines and recommendations on robust and nexus–friendly practices, interventions, packages and strategies Nexus investment pathways
Policy and Governance	Poor horizontal and vertical coordination between institutions, Political economy and incompatibility of current institutional structures, Asymmetries in nexus starting points, Cross–sectoral, cross–disciplinary and intra–entity planning, Funding restrictive and specific to individual sectors. Incoherence in WEF–related policy and governance	Policy and institution coherence	Dialogues and co–planning committees Policy analysis for coherence and harmonization	Cross–sectoral, cross–disciplinary and intra–entity planning Coherent and harmonized WEF– related policy and governance
Capacity Development	Complexity and knowledge gaps: Lack of awareness and knowledge	Curriculum for WEF nexus	Capacity building at all levels	Enhancing understanding of the WEF nexus

## Impact statement

As the water–energy–food (WEF) nexus approach grows and expands, there is a need to apply the nexus to implement technical solutions, resource management, and policy development. Previous studies have comprehensively discussed WEF nexus tools (frameworks, discourses and models) without linking them to applications. This review focused on WEF nexus applications to identify how WEF nexus tools have been applied to facilitate knowledge generation and decision-making in the Global South and some opportunities and challenges arising from these efforts. The review synthesised valuable information on how the nexus tools can generate more knowledge on resource utilisation, especially in constrained environments. Optimistic opportunities for applying nexus approaches to solve real problems and inform policy decisions are provided. The review also reveals that WEF nexus approaches are wider than water, energy and food. There are possibilities of extending to address other global challenges such as climate change, environmental (and ecosystem) degradation, land scarcity, human health, and livelihoods. While there are concerns about data scarcity and scale mismatch when applying nexus methodologies in solving problems, we identify studies that have overcome these hurdles with acceptable results. The review will be of value to scientists and practitioners as it outlines recommendations towards operationalising the WEF nexus approach.
