# Supplementary material for: Review of water–energy–food nexus applications in the Global South
Source: Camb Prism Water. Author manuscript; Available in PMC 2025 Apr 3. (PMC7617535; doi:10.1017/wat.2024.8)
Supplement: supplementary material 1 [file EMS203570-supplement-supplementary_material_1.docx]

**Supplementary Table 1:** Manuscripts used to compile the review

| **Aims and objectives** | **Category** | **References** |
| --- | --- | --- |
| Quantify the environmental value of preserving WERPA by applying the water–food–energy nexus approach and the concept of nonmonetary opportunity cost. | Decision support | Abdelhady et al., 2017 |
| To optimize biofuel supply chain based on Jatropha Curcas L. Oil and used cooking oil (UCO) by considering the nexus between energy, water, food, waste, and land (EWFWL) in order to protect natural resources and create environmental security. | Decision support | Afkhami and Zarrinpoor, 2022 |
| Optimal allocation of land and water resources in the upper Blue Nile (UBN) basin. | Decision support | Allam and Eltahir, 2019 |
| To quantitatively explore various potential solutions and to study four key scenarios that were jointly formulated with the stakeholders | Decision support | Almulla et al., 2022 |
| To design a sustainable supply chain of highly perishable agricultural product (strawberry). | Decision support | Baghizadeh et al., 2021 |
| To simulate the water-energy-food nexus in the Urmia Lake Basin as a holistic multi-sectoral system and to assess the impacts of proposed lake restoration measures, especially looking for trade-offs. | Decision Support | Bakhshianlamouki et al., 2020 |
| To quantify and evaluate the interlinkages of WEF-nexus in transboundary river basins under different system operation settings | Decision support | Basheer et al., 2018 |
| Explore complex non-linear decisions in land use allocation, focusing on the competition between cash and food crops. | Decision Support | Bazzana et al., 2020a |
| To show the relevance and importance of taking into account energy constraints during decision-support on optimal water allocation across a river basin and thus proving the relevance of integrated economic-water-energy management | Decision support | Bekchanov and Lamers, 2016 |
| Examined policy debates related to land, food and water use in sugarcane ethanol production, and the challenges to integrated land–water–energy-food policies. | Decision support | Benites-Lazaro et al., 2020 |
| To inform the sustainable development planning process in the water and energy sectors for both developed and developing countries. | Decision support | Chai et al., 2020 |
| To outline how Nexus Webs can be developed and used to change the decision context to consider options for more socially inclusive and equitable use of water resources. | Decision support | Colloff et al., 2019 |
| To unpack the WEFE nexus of agricultural development in water-stressed regions engaged in export production to meet increasing global food demands. | Decision support | Correa-Cano et al., 2022 |
| To assess for neglected territories in urban areas, considering the residents’ choices and perceptions | Decision support | De Souza et al., 2022 |
| To compare eight Nepalese power development scenarios based on five nexus objectives: minimization of power deficit, maintenance of water availability for irrigation to support food self-sufficiency, reduction in flood risk, maintenance of environmental flows, and maximization of power export | Decision support | Dhaubanjar et al., 2017 |
| Basin-scale assessment of the sustainable hydropower potential by integrating considerations of the water-energy-food nexus, disaster risk, climate change, environmental protection, and socio-economic preferences. | Decision support | Dhaubanjar et al., 2021 |
| To compare three policies—a simple adaptive approach, adaptation with free water to indigent households, and water supply augmentation—and assess their ability to provide reliable FEW services to the different stakeholders under four different climate scenarios, representing moderate to severe amounts of warming. | Decision Support | Ding et al., 2021 |
| To assess water and energy use intensity in irrigated areas of the Karshi Steppe of Central Asia that are supplied by pumping water uphill (lift-irrigated) from the underlying river. | Decision support | Djumaboev et al., 2019 |
| To investigate the future of the WFE nexus with and without the Grand Ethiopian Renaissance Dam (GERD) during filling and subsequent operation using basinwide stochastically generated flows. | Decision support | Elsayed et al., 2020 |
| Explores cooperation pathways in transboundary river basins using a nexus-based approach while considering reservoir system operation and coordination under variable hydrological conditions. | Decision support | Elsayed et al., 2022 |
| To provide a means of decentralized cross-sectoral coordination in the situation of asymmetric information | Decision support | Ermoliev et al., 2022 |
| To estimate the groundwater and energy consumption for cultivating five major crops between 2017 and 2019 at three distinct spatial scales | Decision support | Gaddam and Sampath, 2022 |
| To better understand and aid management of regional FEW nexus systems under uncertainty. | Decision support | Gao et al., 2021 |
| To propose an approach where the best possible (optimized) implementations of different river basin development scenarios are assessed by comparing their WEFE sector trade‐offs. | Decision Support | Geressu et al., 2020 |
| To modify groundwater governance based on a participatory, water-food-energy (WFE) nexus. | Decision support | Ghafoori-Kharanagh et al., 2021 |
| To highlight advantage and minimise damaging and unsustainable outcomes through informed decisions regarding trade-offs inclusive of economic, ecological and equity considerations. | Decision support | Gomo et al., 2018 |
| To develop sustainable development of irrigated agricultural production and distribution of resources | Decision support | Guo et al., 2022 |
| Through the Comprehensive Index Evaluation System of adaptability, to systematically evaluates the adaptability of the Yangtze River Delta the Water-Energy-Food System from 2010 to 2019. To provide policy recommendations for the adaptive optimization of the Water-Energy-Food System in the Yangtze River Delta, in expectation of promoting regional sustainable development. | Decision Support | Han and Ma, 2021 |
| Optimal allocation of electrical energy, water, and land resources for a region in the north of China. | Decision support | Hassas et al., 2021 |
| To outline the optimal development in the basin to ensure food and energy security with a sustainable development approach. | Decision support | Hatamkhani and Moridi, 2019 |
| To assess establishment of clean energy-driven desalination-irrigation (CEDI) systems, requiring multi-criteria group decision-making | Decision support | Hu et al., 2020 |
| To raise urban food security with less use of public water and energy in food production, through utilizing green water and energy for sustainable management. | Decision support | Huang and Chang, 2021 |
| For planning the WEFN system of Henan Province, China | Decision Support | Huang et al., 2022 |
| To identify and help decision-makers visualise reservoir management strategies which result in the best possible (Pareto-optimal) allocation of benefits between sectors; to show how trade-offs between achievable benefits shift with the implementation of proposed new rice, cotton and biofuel irrigation projects. | Decision Support | Hurford and Harou, 2014 |
| To examine two potential operation modes of the dam: Energy Mode (ensuring Tajikistan’s hydropower needs) and Irrigation Mode (ensuring water for agriculture downstream). | Decision support | Jalilov et al., 2016 |
| To support regional crop-biomass coproduction management under uncertainties while accounting for the complex interactions of FEW nexus. | Decision support | Ji et al., 2020 |
| To assess the sustainable development of bioenergy from cassava, considering landuse change and climate change with a biogeochemical process model within the “water-energy-food” nexus framework. | Decision support | Jiang et al., 2018 |
| To apply the water-energy-food nexus index (WEFNI) as a new management perspective at the farm level. | Decision support | Karamian et al., 2021 |
| Evaluate and compare the impacts of alternative development trajectories pertaining to agriculture, energy and environment for a case-study location, the Lake Tana Subbasin, with a focus on current national plans and accounting for cross-sector interlinkages and competing resource use: the food-energy-environment nexus. | Decision support | Karlberg et al., 2015 |
| To simulate the impacts of water resource management decisions that affect the food–water–energy–environment (FWEE) nexus | Decision Support | Khan et al., 2017 |
| To investigate the responses of water resources in Indus River under different emission scenarios in future and the response of Tarbela reservoir to the available water. Provides valuable information to assist policy makers and management in the area of agriculture, Tarbela Reservoir management, energy production and related areas in the future. | Decision support | Khan et al., 2017 |
| For efficient resources use technology or resource management scenarios to meet or improve urban WEF demands for Addis Ababa city. | Decision support | Kitessa et al., 2022 |
| Establishing to what extent weighting approaches to constrain climate uncertainty might affect the nature of decision-relevant climate impact information. | Decision support | Kolusu et al., 2021 |
| To identify the optimum allocation of 13 sources of treated wastewater to be used in cultivating alfalfa, achieving reductions in greywater footprint, energy for transportation, and carbon footprint. | Decision support | Lahlou et al., 2020 |
| Optimally allocating both surface water and groundwater to different growth periods of rice in different subareas in Heping irrigation area, Qing’an County, northeast China | Decision Support | Li et al., 2019 |
| To aid in allocation of resources toward the sustainable management of agricultural water, food, and energy nexus under uncertainty. | Decision support | Li et al., 2019 |
| Assess approach (input/output relationship) for sustainable bioenergy production in agricultural systems. | Decision support | Li et al., 2020 |
| To seek a sustainable cropping system to balance crop production while reducing energy consumption and water depletion. | Decision support | Li et al., 2021 |
| To assess the efficiencies of W-E-F nexus in 13 selected cities of Jiangsu Province in China, where W-E-F nexus is innovatively decomposed into three stages, “W-E,” “WE-F,” and “WEF.” | Decision support | Li et al., 2021 |
| To quantify the interactions and feedbacks within water, land, food and energy subsystems; provide trade-offs among water and energy utilization efficiency, economic benefits and environmental protection in agroforestry systems; and generate optimal policy options among water and land resources for different crops and woodlands in different regions under different climate change patterns. | Decision Support | Li et al., 2021 |
| To simulate changes in the FEW demand and supply from 2000 to 2050 in Beijing | Decision support | Li et al., 2021 |
| For the synergistic regulation of water, land, energy, and carbon emissions in a circular agricultural system by balancing water supply and demand, land allocation, electricity consumption, and economic upgrading principles. | Decision support | Li et al., 2022 |
| To evaluate the changes in FEW resource security along with the development of the urban social-economic system; and to explore possible solutions to enhance urban FEW security by balancing resource supply and demand and increasing synergies between components using scenario analyses. | Decision support | Li et al., 2022 |
| To conduct LCA of a small-scale WWEF system built for Brazilian rural areas and identify the environmental hotspots to provide evidence-based information to support decision support in treating waste processes for cassava starch and flour industry. | Decision support | Lin et al., 2021 |
| To generate optimal security management policy for a water–energy–food nexus system of the urban agglomeration under multiple uncertainties. | Decision support | Liu et al., 2020 |
| To analyze the interactions among WFE nexus in agricultural watersheds, which considers various scenarios such as water-saving irrigation and drought. | Decision support | Liu et al., 2022 |
| To assess resource utilisation and performance in southern Africa, in the context of achieving the 2030 global agenda of SDGs, that is, how best to build resilient rural communities and enhance sustainable rural livelihoods. | Decision support | Mabhaudhi et al., 2019 |
| To investigate and quantify PV generation potential, without significantly harming agriculture output | Decision Support | Malu et al., 2017 |
| Propose stakeholder identification and categorization for a nexus assessment of the Ribeirão das Lajes reservoir whose resources are threatened by anthropic action and climate change. | Decision support | Melloni et al., 2020 |
| To maximise the water-energy-food index, maximizing gross margin, minimizing the use of fertilizers and chemical pesticides, regarding the constraints of balancing groundwater resources to reduce pollution and environmental destruction for the optimal and sustainable management of the resources in the agricultural sector. | Decision support | Mirzaei et al., 2022 |
| To promote sustainable agricultural practices and natural resources preservation in the rural south zone of São Paulo | Decision support | Moreira et al., 2022 |
| To allocate scarce water resources more efficiently by integrating different scientific perspectives and harmonizing the needs of energy and food production | Decision support | Müller-Mahn et al., 2022 |
| To investigate the effects of anticipated dynamics of integrated water and energy sectors in the next two decades. | Decision support | Naderi et al., 2021 |
| To assess the current strategies associated with EWF-related activities, and to direct policymakers in the establishment of future national priorities programs that meet the needs of the country from energy, water and food resources for an improved social welfare and enhanced economic achievements through ensuring high performance in the three pillars of efficient resource management: resilience, sustainability, and security. | Decision Support | Namany et al., 2021 |
| To develop a robust and systematic methods to derive trade-offs for land use decision-making. | Decision support | Nie et al., 2019 |
| Leverages on multi-objective sustainable land use allocation models, integrated with energy and water resource allocation challenges in the food sector, taking cognisance of the FEW-N. | Decision support | Ogbolumani and Nwulu, 2021 |
| To address natural resource allocation for food and energy security. | Decision Support | Ogbolumani and Nwulu2021 |
| Formulation of a Climate-resilient water safety plans in Leh Town, India, a city with rapid development and population growth located in the Himalayas—one of the most sensitive ecosystems to climate change. | Decision support | Páez-Curtidor et al., 2021 |
| Estimating a more diverse and sustainable electricity mix so that risks related to overreliance in just one generation source are mitigated. | Decision support | Paim et al., 2019 |
| To determine the impacts of WEF related projects on transboundary watershed | Decision support | Payet-Burin et al., 2019 |
| To explore: (1)What are the benefits of performing infrastructure planning in a “nexus” framework (considering the water, energy, and food sectors jointly) vs. In “silo” frameworks (considering sectors separately)? (2) Does the joint evaluation of intersectoral investments lead to higher synergies and less tradeoffs between the selected investments? (3) Does the nexus framework identify risks that are otherwise not perceived in a silo framework? | Decision support | Payet-Burin et al., 2021 |
| Using a large range of scenarios, we investigate in which cases the perfect foresight assumption affects the economic evaluation of two hypothetical projects. Finally, to perform the same analysis for the economic evaluation of hydropower expansion, irrigation development, and an environmental flow policy on the Zambezi River Basin | Decision support | Payet-Burin et al., 2021 |
| To illustrate how scarcity in water resources affects the tradeoffs between food and energy and explore the political and social constraints that can move production away from what is feasible technically. | Decision support | Perrone and Hornberger, 2016 |
| To explore potential approaches to coordinating the FEW nexus in grain production based on a conceptual framework that incorporates the impact of rural livelihood transitions and farmland resource constraints. | Decision support | Qi et al., 2021 |
| To plan a groundwater, energy, and food nexus system | Decision support | Radmehr et al., 2020 |
| Develop a common ground for strengthening cross-sectoral coordination in planning and prioritizing activities and for resource allocation and investment, using South Asia as an example. | Decision support | Rasul and Neupane, 2021 |
| For planning 14 crops planted in orchard, irrigated farms, and rain-fed farms, between 2006 and 2014, and targeting water-energy-food nexus index (WEFNI) maximization. | Decision support | Sadeghi et al., 2020 |
| To foster an integrated programme of sustainable food production and water provision in the framework of the water-energy-food nexus. | Decision support | Saladini et al., 2018 |
| Identifies and evaluates unrealised complementarities between nexus governance and water diplomacy, and discusses the benefits of integrating both for improved transboundary basin management. | Decision support | Salmoral et al., 2019 |
| To examine a pairwise comparison of water and energy sources, sanitation facilities and food accessibilities in six sampling communities and tracking progress towards achieving the sustainable development goals (SDG) at a local scale using nine specific SDG indicators. | Decision support | Sani and Scholz, 2021 |
| To optimize the balance between CO2 emissions, water consumption, energy consumption, food production, cost, and benefits during the cultivation period and improve the synergistic benefits of the WFE Nexus in coming years. | Decision support | Saray et al., 2022 |
| Analyze how demand and supply for biomass energy in Malawi will evolve in the coming years and which policy measures could ensure a sustainable biomass energy sector | Decision Support | Schuenemann et al., 2018 |
| To demonstrate how the nexus approach, when embedded in a farm budget model, can make a notable contribution to understanding how a changed relationship among water, energy and food affects farm profitability. | Decision Support | Seeliger et al., 2018 |
| Optimal planning of resource allocation in Malaysia’s agricultural sector. | Decision support | Siah and Zabiri, |
| Explore the costs and potential water savings of 24 combinations of water conservation measures in the Rufiji basin, Tanzania. | Decision support | Siderius et al., 2022 |
| To quantify and monitor the WEF nexus interlinkages at a national level. | Decision support | Simpson et al., 2022 |
| To generate an optimum debottlenecked Palm Oil Based Complex flowsheet with maximised gross profit (GP) and net energy enhancements subjected to environmental constraints in terms of water and GHG footprints | Decision support | Tan et al., 2021 |
| To assess the potential of small-scale AD considering the context of WEF nexus to identify economically feasible solutions for smallholder farmers and evaluate the financial- and broader economic benefits for major stakeholders, which will help to support bioenergy production and resource circularity within agricultural systems. | Decision support | Tolessa et al., 2022 |
| To identify efficient management alternatives to hypothetical agricultural scenarios | Decision support | Udias et al., 2018 |
| Apply a holistic three-fold scheme that synergistically optimizes the benefits of the Water-Food-Energy (WFE) Nexus by integrating the short/long-term joint operation of a multi-objective reservoir with irrigation ponds in response to urbanization. | Decision support | Uen et al., 2018 |
| To determine the allocation of water for food production and urban use in Kenya; and the production and allocation of energy for food production in Hyderabad, India. | Decision support | Villamayor-Tomas et al., 2015 |
| Implementing sustainable transformative solutions in transboundary river basins. | Decision support | Wada et al., 2019 |
| Application of an integrated decision-support tool based on the resilience.io platform, which provides a series of modules to allow forecasting of socio-demographic scenarios, simulating spatio-temporal activities, and planning investment and operational strategies to meet the Sustainable Development Goals (SDGS). | Decision support | Wang et al., 2018 |
| To construct the supply-demand risk assessment framework, identify the risk factors during different periods in the Beijing-Tianjin-Hebei (BTH) region, and simulate the probability of supply-demand risk under different scenarios. | Decision support | Wang et al., 2021 |
| To improve the BTHWEF nexus synergies through resolving resource supply–demand imbalance and promoting sustainable social development. | Decision support | Wang et al., 2022 |
| To explore the impacts of real policies designed by different government departments on the WEFN system | Decision Support | Wen et al., 2022 |
| Understanding how energy, water, and land (EWL) systems may interact with one another and these strategies is advantageous for national planning, particularly for identifying synergistic interventions (e.g., investments in infrastructure, policies, and institutions). | Decision support | Wild et al., 2020 |
| To develop different circular economy scenarios associated to FEW management. UCEC v1.0 uses Beijing data as test case. | Decision support | Xue et al., 2018 |
| To mobilize natural and social resources in urban spaces with integrated technology and knowledge in order to uncover and carry out FEW management innovations. | Decision support | Yan and Roggema, 2019 |
| To assess sweet sorghum-based ethanol potential in China in compliance with the Water–Energy–Food nexus principles. | Decision Support | Yan et al., 2018 |
| To quantify the sustainability of water availability in a conceptually coherent way for trade-offs analysis (at basin-wide, national, and regional levels) to support investment decisions. | Decision support | Yang et al., 2018 |
| Explore the divergence of assessment results when weak and strong sustainability criteria are applied, and consider the practical ramifications of these concepts using Jiangsu Province, China, as a case study. | Decision support | Yi et al., 2020 |
| For planning the WEFN system of Henan Province (China) in which three decision targets (i.e. System benefit, water resources utilization and energy consumption) are included | Decision Support | Yu et al., 2020 |
| (1) to compose a primary list of urban sustainability indicators for comparative basis, (2) to examine the variations of indicators applied in individual cities implementing urban sustainability, and (3) to identify possible solutions for urban sustainability practice. | Decision support | Yuan et al., 2021 |
| To seek for coordinated water and land resources allocation solutions, and consequently achieve SDGs concerning efficiency-equity tradeoff, food security, energy security, and farmland ecosystem sustainability. | Decision Support | Yue et al., 2021 |
| To assess the impacts of reservoir operation on the WEFS nexus | Decision Support | Zeng et al., 2022 |
| To explore the seasonal fallow strategy as a measure to limit shallow groundwater withdrawal in a well-irrigated plain of the Haihe River basin of China via (1) simulating the shallow groundwater variation, soil water balance, and crop yield changes under the designed fallow scenarios using a distributed hydrological model; (2) evaluating the effects of different fallow schemes on shallow groundwater conservation, crop production, and energy consumption through quantitative indices with regard to the WFEN perspective; and (3) optimizing the fallow scheme pattern in the study area that can simultaneously achieve shallow groundwater equilibrium, minimize crop yield reduction, and improve water and energy use efficiency. | Decision support | Zhang and Ren 2021 |
| To identify the critical factors/nodes of cross-sectoral or cross-regional WEF resources trade for helping scientific and rational management on supply-demand of WEF resources to cope with the continuous growth of WEF resource demand in the future. | Decision support | Zhang et al., 2021 |
| To quantify the WEF nexus and reveal the complex interactions. Results could support cross-regional joint management of WEF resources and WEF-related sdgs in Guangdong, Hong Kong, and Macao regions. | Decision support | Zhang et al., 2022 |
| To assess the hydrologic impacts of land use transformation, in particular replacing agricultural land with urban land. | Decision Support | Zhao etal2017 |
| To optimize floating PV deployment on irrigation ponds and existing hydropower | Decision Support | Zhou et al., 2020 |
| To plan agricultural water, energy and food (WEF) as well as crop area management. | Decision support | Zuo et al., 2021 |
| To investigate the long-term profitability of large hydropower stations by considering the nexus between the environmental, economic, and social aspects | Decision support | Tajziehchi et al., 2022 |
| To measure the WEF security nexus, where the optimal distribution of resources is evaluated through different allocation schemes | Decision support | Cansino-Loeza et al., 2022 |
| To incorporate the photovoltaic panel rainwater harvesting system into the agricultural WEF nexus, providing a decision-making framework that exploits and conserves resources in parallel, while contributing to economic benefits. | Decision support | Ye et al., 2023 |
| To designate optimal tillage and irrigation management patterns for a winter wheat-summer maize double cropping system | Decision support | Zhang et al., 2022 |
| Six future scenarios were designed, and the development trends of the WEFN system under different development scenarios were simulated to explore the impact of different policies on the WEFN. | Decision support | Gao et al., 2023 |
| To extract the resource allocation pattern based on the goals of the WEFN system and evaluate the cooperative behavior of farmers with this pattern under government’s policy options | Decision support | [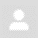](https://www.frontiersin.org/people/u/1797724)   \| [Mirzaei et al., 2023](https://www.frontiersin.org/people/u/1797724) \| \| --- \| |
| To evaluate the economic, environmental, and social feasibility of the closed-loop dairy system. | Decision support | Muell et al., 2022 |
| To evaluate the water–energy–food nexus and also to identify the action required to enhance resource productivities. | Decision support | Chapagain et al., 2023 |
| Help stakeholders visualize trade-offs between competing objectives, understand the implications of interactions across sectoral boundaries, and select the optimal system for their context. | Decision support | Oviroh et al., 2023 |
| To improve water and energy efficiency, simultaneously maximise the WEFNI and minimise agricultural CO2 emissions, and ultimately determine crop production and emissions reduction potential in the TRB. | Decision support | Feng et al., 2024 |
| To propose alternatives to maximize food production by integrating water management and energy generation for the local community's welfare and respecting the biome's specificities. | Decision support | Viana et al., 2023 |
| To evaluate the WEF Nexus sustainability. The Analytical Hierarchy Process (AHP) was employed to identify the most crucial indicators for policymakers | Decision support | Babaei et al., 2024 |
| To effectively redistribute production resources within the agricultural sector. | Decision support | Barzin et al., 2024 |
| To provide adequate decision support for the WEF nexus management in water-scarce areas | Decision support | Sun et al., 2023 |
| To examine the effects of future growth of a cascade hydropower system with reservoir storage on the mainstream of the YB River by simulating hydropower generation, reservoir operation, agricultural irrigation, and flood mitigation in the YB River basin from the perspective of WEF nexus. | Decision support | Lyu et al., 2023 |
| To identify efficient operation plans for large dams on the Blue Nile for alternative cropping patterns in expanded irrigation areas in Sudan that minimize tradeoffs across water, energy, and food objectives. | Decision support | Basheer et al., 2024 |
| To select the most robust renovation plan for a hydropower system in the basin by creating a robust and objective weighting mechanism to quantify the role of each sector in the decision-support process | Decision support | Enayati et al., 2021 |
| To simulate China’s water-energy-food system, and scenarios of the “two-child” policy and trade friction | Decision support | Jin et al., 2019 |
| To characterise WEFN systems, prescribe the WEFN problem, explore its causes and consequences, and identify effective measures and policies for alleviating conflicts. | Decision support | Zhang et al., 2021 |
| To design a sugarcane-based bioenergy supply chain network under Water-Energy-Food-Land (WEFL) nexus considerations. | Decision support | Abdali et al., 2021 |
| Analyses the impacts of first-generation sugarcane expansion in the Paranaíba basin (Goiás State), focusing on how future demand for ethanol could affect local resources availability. | Decision support | Bellezoni et al., 2018 |
| To explore the robustness of adaptation options and pathways against future climate and socio-economic uncertainties in the Cauvery River Basin in Karnataka, India | Decision support | Bhave et al., 2018 |
| To conduct an analysis of stakeholder and partner qualitative interview data collected within the LIVES (Linked Indicators for Vital Ecosystem Services) project to explore three examples how we engaged with power dynamics in the course of the research | Decision support | Bréthaut et al., 2019 |
| To demonstrate the WEF Nexus Tool utility as a decision-support guide in a case study for Qatar’s food security | Decision support | Daher and Mohtar, 2015 |
| Evaluate in a reservoir-canal command for optimal water and energy uses and, subsequently, compared with the conventional models of Net-Economic Return (NER), Water-Food (W-F) nexus, and Energy-Food (E-F) nexus based approaches using multi-criteria decision support (MCDM) analysis. | Decision support | Das et al., 2021 |
| To propose an optimal cropping pattern that considers water, energy and economic parameters. | Decision support | EL-Gafy et al., 2017 |
| To present projected hydrological changes caused by multiple drivers, namely climate change, large-scale hydropower developments, and irrigated land expansions by 2050s. | Decision support | Hoang et al., 2019 |
| To plan WFE nexus system associated with hierarchical structure and random uncertainty | Decision support | Ma et al., 2020 |
| Urban level assessment of collective FEW security in Nagpur, India | Decision support | Morey et al., 2022 |
| To holistically assess resource availability, distribution, use and management at a local level in Sakhisizwe Local Municipality, South Africa. | Decision support | Nhamo et al., 2020 |
| Discusses the potential hazardous events associated with a drinking water supply and management strategies in the case of Mysuru city, India to realize water security through integrated modeling approaches. | Decision support | Pallavi et al., 2021 |
| To uncover unexpected high-order effects of policy objectives, and could lead to policy makers exploiting synergies and avoiding or mitigating detrimental impacts across the nexus. | Decision support | Purwant et al., 2019 |
| To evaluate the effects of future climate (2040–2069), with and without irrigation expansion, on water levels and flows in an agricultural watershed in low-storage crystalline aquifer region of south India. | Decision support | Sishodia et al., 2018 |
| To investigate the relationship between water, food, and energy (W–E–F) in different regions of China to provide suggestions for sustainable and stable regional development of these resources. | Decision support | Sun et al., 2021 |
| To perform optimal retrofit of palm oil mill into proposed palm oil-based complex with optimal EFEW nexus contributions. | Decision support | Tan et al., 2021 |
| To quantify resource consumption and production and, consequently, the future resource sustainability on a national scale | Decision support | Wicaksono et al., 2019 |
| Examines water demand for the agriculture and energy sectors in the YRB until 2030. It then analyzes whether and to what extent various policy options, especially agricultural water savings and inter-basin water transfers, can meet the water demand for both sectors without draining the YRB | Decision support | Xiang et al., 2017 |
| To generate optimal water and land use alternatives under different hydrological years | Decision support | Zhang et al., 2021 |
| To calculate the allocation scheme of water resources supply In each province of China | Decision support | Zhang et al., 2022 |
| To assess the future land and water demand triggered by sugar crops expansion from the food-energy-water/land nexus perspective. | Decision support | Zhong et al., 2021 |
| To seek for coordinated resources allocation solutions considering resources input reduction, agricultural wastes recycle, global warming mitigation and socio-economic security, consequently promote sustainable circular agricultural development. | Decision support | Yue et al., 2022 |
| To analyze the effects of global warming scenarios on the co-benefits between hydropower and irrigation. | Decision support | Meng et al., 2021 |
| A comprehensive assessment of Afghanistan’s water resource management, including the current state, challenges, opportunities, and way forward | ecision support | Shams et al., 2022 |
| To qualitatively interrogate and untangle how food production and the anticipated outcomes of agricultural technological interventions can be affected by interlinkages involving water, energy, and climate. | Understanding | Adebiyi et al., 2020 |
| Analyzed the environmental injustice issue relationships of the supply and production of water, energy, and food in the Sao Paulo Macrometropolis, Brazil. | Understanding | Amaral et al., 2021 |
| To quantitatively evaluate the performance of surface water distribution systems based on the water-food-energy nexus in the Rudasht irrigation network in Isfahan by upgrading the operation method to the improved manual operation, decentralized automatic operation, and centralized automatic operation methods. | Understanding | Bayat et al., 2022 |
| To study the impact that water canals and electric grid development have on the Water-Energy-Food (WEF) nexus in a rural area. | Understanding | Bazzana et al., 2020 |
| To examine the changes in social policy debates related to ethanol production in Brazil and its relationship with climate change and food security. | Understanding | Benites-Lazaro et al., 2018 |
| To explore the interactions between development pathways and climate change uncertainty, for a least developed country (LDC) context in Africa; Malawi. | Understanding | Bhave et al., 2020 |
| To explore the effect of carbon credit price variations, feed-in-tariffs and agricultural intensification (thereby illustrating the framework's relevance to policy and investment decision-making), as well as the impact of climate change on inland waters and energy production | Understanding | Bieber et al., 2018 |
| To answer three main questions: (a) How much benefit could the energy sector potentially lose when water is shared economically with the agriculture sector? (b) How much benefit the agricultural sector brings into the system when water is shared with the energy sector? (c) How likely are those benefit changes, considering hydrologic variability? | Understanding | Bof et al., 2021 |
| To promote debate and broaden understanding of currents trends related to the water-energy-food nexus through an exploratory modelling analysis | Understanding | Borgomeo et al., 2018 |
| To comprehensively evaluate the WEF system security of the CPEC for the period 2000–2016. | Understanding | Cao et al., 2022 |
| Uncover the scarce water footprint and the interregional virtual water flows associated with sugarcane-derived biofuel production driven by domestic final consumption and international exports in 27 states in Brazil. | Understanding | Castillo et al., 2017 |
| To quantify causality relations between water, energy, food and economy, society, environment | Understanding | Chai et al., 2019 |
| To study the water–energy–food symbiosis of the Yangtze River. | Understanding | Chen and Chen, 2021 |
| To calculate the symbiotic security index, determine the water security state and pre-warning level and analyze the causes of water security problems | Understanding | Chen and Chen, 2021 |
| To evaluate the security and the main driving factors affecting the synergistic security of the water-energy-food system | Understanding | Chen and Xu, 2021 |
| To calculate the regional total factor productivity (TFP) and the to investigate the change trend of the TFP from 2007 to 2016 based on understanding the water–energy–food nexus. | Understanding | Chen et al., 2019 |
| Investigate the food-energy-water nexus (FEW nexus) for a small and multi-sector island city, Kinmen | Understanding | Chen et al., 2020 |
| To examine the interdependence of the three natural resources, and the LCA was used to evaluate the environmental impacts of the WEF system. | Understanding | Chen et al., 2020 |
| To demonstrate a multi-objective optimization study of a water–energy–food system. | Understanding | Chen et al., 2020 |
| To study the specific supply-and-demand mechanism of water resources in each subsystem. | Understanding | Chen et al., 2020 |
| To investigate the potential and the global impact of biodiesel production on the EWF nexus. | Understanding | Chong et al., 2021 |
| Based on WEF Nexus quantification, combining traditional agricultural water-saving and water footprint theory, the sustainable development level (SDL) of water acquisition-transfer-consumption process for pumped irrigation systems was analyzed, using the principal component analysis (PCA) and taking the Lianshui Irrigation District (LID) in eastern China as a case study | Understanding | Cui et al., 2022 |
| To assess the water demand and energy footprint of irrigated production in the region. | Understanding | Daccache et al., 2014 |
| To examine how nutrients from the wastewater that is disposed to Lake Chivero can assist in contributing to the food production in the Harare city region. | Understanding | Das and Chirisa, 2021 |
| To understand better the challenges of peri-urban territories towards social justice, with a view to Brazilian agrarian reform areas. | Understanding | De Melo et al., 2020 |
| To provide insight in some of the current water resources problems focusing on the Mediterranean—as a WEFE hotspot in Europe -, with areas of groundwater depletion, and rivers with low flow issues. | Understanding | De Roo et al., 2021 |
| To better understand the hidden links between urbanization and FEW systems | Understanding | Deng |
| To quantify and capture the critical final demand categories that have an important impact on embodied water-energy food uses in China | Understanding | Deng et al., 2020 |
| To quantify and capture the critical categories in final demands that have an important impact on embodied FEW uses in murs and identify the change features at a temporal scale. | Understanding | Deng et al., 2021 |
| To calculate the embodied FEW flows in the Bohai MUR, identify the embodied FEW flow changes in the economy and quantify the contributions of the driving forces to these changes. | Understanding | Deng et al., 2022 |
| Basin-scale assessment of the sustainable hydropower potential by integrating considerations of the water energy-food nexus, disaster risk, climate change, environmental protection, and socio-economic preferences. | Understanding | Dhaubanjar et al., 2021 |
| To study the FEW nexus connecting the agricultural, urban, and hydroelectric generation sectors. | Understanding | Ding et al., 2021 |
| Quantify the effects of reservoir operation on hydropower generation, irrigated crop production and fisheries yield in the Tonle Sap lake through a novel hydro-economic model at the whole basin scale. | Understanding | Do et al., 2020 |
| Exploring the urban water-energy-food nexus under environmental hazards within the Nile | Understanding | Elagib et al., 2021 |
| Analysis of dynamic behaviour, focusing on joint water–energy–food interactions stemming from crop production and consumption at the national level under different scenarios and alternatives. | Understanding | El-Gafy et al., 2016 |
| To analysis the water–food–energy nexus of the crop production system at the national level and carry out a quantitative assessment | Understanding | El-Gafy, 2017 |
| Takes six counties (districts) of the Zhangye city as typical cases and calculate the FEW nexus indexes from a more intuitive and simple perspective in 2005–2015. | Understanding | Feng et al., 2020 |
| To comprehensively evaluate the water and energy consumption, water and energy productivity, and the WEFNI of the main crops (rice, wheat, maize and cotton) in the TRB from 1990 to 2019. | Understanding | Feng et al., 2022 |
| The case study involves various chemical processes operating in the State of Qatar, which is evaluated to ascertain resource efficiency enhancement possibilities across various scenarios, and considers multiple objectives to capture the trade-offs between minimising the total annual cost and environmental emissions. | Understanding | Fouladi et al., 2021 |
| To emphasize the shift to small-scale and highly localized WEF systems and the product and service system based on the satisfaction unit, i.e., designing SPSS applied to Distributed WEF Nexus. | Understanding | Gao et al., 2022 |
| To examine the performance of Conservation Agriculture Sustainable Intensification (CASI) -managed and traditional Conventional tillage managed cropping systems; to assess the effectiveness of CASI under different cropping systems and across different regions of the and to describe the enabling social and policy frameworks which supported the uptake of CASI. | Understanding | Gathala et al., 2020 |
| Understanding the nexus among water, energy, and food for bioethanol in Pakistan and its environmental implications in a life cycle perspective. | Understanding | Ghani et al., 2019 |
| To evaluate the water-energy-food (WEF) system security and clarify the main factors affecting WEF development, system state and social response. | Understanding | Gu et al., 2022 |
| To assess the water–energy–food nexus (WEFN) in the sugarcane production sector of Ethiopia. | Understanding | Hailemariam et al., 2019 |
| To consider multiple combinations of dimensions to assess the performance of China’s FEW nexus system from 2005 to 2017; the dimensions included the economic benefits, social benefits and negative environmental impacts. | Understanding | Han et al., 2020 |
| To assess the degree of WEF security in the five Central Asian countries during 2000–2014, analyse the spatiotemporal variations in WEF security, reveal the relationships among the three sectors, and identify the factors that positively or negatively determined WEF security | Understanding | Hao et al., 2022 |
| To evaluate intensities between related factors in the local WEF-Nexus using the panel data of China’s 30 provinces from 2005 to 2016 | Understanding | Huang et al., 2020 |
| To analyse the input–output efficiency of the water-energy-food nexus by considering production-based intensity, consumption-based intensity, and the quantity index system. | Understanding | Huang et al., 2021 |
| To investigate the impact of four possible scenarios: market force, fortress world, great transition and policy reform | Understanding | Hussien et al., 2017 |
| Analyzed the ecological balance of human activities in Rwanda and how policymakers have increasingly emphasized on energy–water–food nexus sectors separately without integrating land usage and population growth which poses an even more critical situation if left unattended to. | Understanding | Imasiku and Ntagwirumugara, 2019 |
| To identify the most economically productive mix of expanded reservoir storage for economic benefit sharing to occur, in which economic welfare of all riparians is improved. | Understanding | Jalilov et al., 2018 |
| To quantitatively evaluate regional WEF synergy development in the Ningxia Hui Autonomous Region | Understanding | Jiang et al., 2018 |
| Assess agricultural transformation and energy transition in Rwanda, and to highlight the trade-offs and synergies | Understanding | Johnson and Karlberg, 2017 |
| Conceptualize natural resource, analyse nexus policy coherence priorities, power and political perspectives. | Understanding | Johnson et al., 2018 |
| To explore the potential co-evolution of agricultural transformation and energy transition in Rwanda, and to highlight the trade-offs and synergies between them | Understanding | Johnson et al., 2018b |
| To elaborate on smart greenhouses in the food-energy water nexus | Understanding | Karanisa et al., 2022 |
| To contribute to the combination of the two previously mentioned FWE nexus approaches—integrated systems modeling and the co-production of FEW nexus knowledge. | Understanding | Karutz et al., 2022 |
| Investigated water, energy, and food nexus dynamics for the trades of a few major crops, specifically considering Saudi Arabia. | Understanding | Kashifi et al., 2022 |
| The impact of the water–energy–food nexus on the economy and the value of efficiency | Understanding | Ke Zhang et al., 2022 |
| Identify and discuss two parallel dimensions for the nexus, with one focusing on research and analysis and the other on integrated planning and cross-sectoral collaboration. | Understanding | Keskinen et al., 2015 |
| To investigate the simulation of sustainable water resources management to assess the impact of socio-economic development on water, food and energy resources security in Khuzestan Province in Iran. | Understanding | Keyhanpour et al., 2021 |
| Identifies research that is needed to better understand and govern issues related to W–E–F Security Nexus | Understanding | Lawford et al., 2013 |
| Examine how nexus links have been governed, using four medium- to large-scale water infrastructure projects in Laos and Thailand as probes into problematic issues of coordination, anticipation, inclusion, and attribution. | Understanding | Lebel et al., 2020 |
| To investigate W-E-F nexus focusing on the downstream industry that is consumption-oriented. | Understanding | Lee et al., 2021 |
| To link the circular economy discourse with the Urban Nexus. | Understanding | Lehmann, 2018 |
| To evaluate the embodied resource consumption in WEF nexus in Taiwan, and to distinguish resource flow into direct and indirect consumptions. | Understanding | Li and Ma, 2020 |
| Evaluate the WEF-Nexus input-output efficiency with the data envelopment analysis (DEA) model. | Understanding | Li et al., 2016 |
| To examine the ISM analysis framework and urban WEF-nexus conceptual framework. | Understanding | Li et al., 2019 |
| Explores the synergies within the WEF-Nexus in Shenzhen city while using the synergetic model. | Understanding | Li et al., 2019 |
| Examined the interrelationships between land cover change, dam-related flooding and access to safe water via a national-level spatial analysis with local case studies in Malawi and Ghana. | Understanding | Li et al., 2021 |
| To assess the interactions and synergistic relationships among biomass electrical energy, light energy, and hydroelectric energy, clarify the dynamic characteristics of resource allocation and socioeconomic and environmental effects, and capture the high uncertainty in the nexus area. | Understanding | Li et al., 2022 |
| To determine the energy balance, carbon and water footprints (CF and WF, respectively) in 50 farms, organic and conventional, where four MAP species were cultivated; spearmint (Mentha spicata), oregano (Oreganum vulgare), rosemary (Rosmarinus officinalis) and Damask rose (Rosa damascena). | Understanding | Litskas et al., 2019 |
| To evaluate agricultural sustainability in a given region with intensive agricultural practice. | Understanding | Liu et al., 2019 |
| To estimate the Central China region’s energy-food production water footprint (WF) and virtual trade water flow from 2001 to 2016. | Understanding | Lu et al., 2021 |
| To study the water–energy–food linkage in economic system | Understanding | Lui et al., 2017 |
| To investigate the WFE interplays in China concerning irrigated agricultural production and hydropower potential. | Understanding | Lui et al., 2019 |
| Analyzed the urban water-energy-food nexus under environmental hazards | Understanding | Ma et al., 2021 |
| To glimpse the status of each sector regarding its resources and to obtain a general picture about the WEF nexus | Understanding | Mahlknecht and González-Bravo 2018 |
| To examine the baseline and trends of resource security based on the Water-Energy-Food Nexus concept | Understanding | Mahlknecht et al., 2020 |
| Assess and quantify how the nexus solutions deployed in 29 case studies in the Mediterranean region contribute to a potential achievement of SDG targets by 2030 with regard to the sustainability from the perspectives of economy, environment and society. | Understanding | Malag´o et al., 2021 |
| To track the urban EWL flows not only within local but also across regional, national, and even global supply chains from the production- and consumption-based perspectives using the environmentally extended multiscale input output model (EE-MSIO). | Understanding | Meng et al., 2022 |
| Comparing the case of untreated sewage (partly reused in agriculture and the remainder released to the river) versus treated WWTP effluent (partly reused in agriculture and the remainder released to the river) in terms of water-energy-health impacts of urban agriculture. | Understanding | Miller-Robbie et al., 2017 |
| To quantify FEW security and highlights cases in Ecuador, Peru, and Bolivia where national statistics leave out important regional variation. | Understanding | Mohammadpour et al., 2019 |
| To investigate water-energy-food-land nexus (WEFLN) in the Jatiluhur reservoir, the largest reservoir in Indonesia | Understanding | Muhamad, 2020 |
| To assess the ecological sustainability response to the high increase demand for WEF resources in well-developed African countries. | Understanding | Muhirwa et al., 2022 |
| To discuss the suitability and shortcomings of nexus approaches for the analysis of complex socio-ecological transformations. | Understanding | Müller-Mahn and Gebreyes, 2019 |
| To evaluate the contributions of ecosystem-based adaptation(EBA) practices to the water–energy–food (WEF) nexus balance, design practical pathways, and analyze barriers towards achievement of EBA-WEF balance. | Understanding | Muthee et al., 2021 |
| To explore the dynamic relationships between Water-Energy-Food (WEF) resources, carbon-fossil-GHG emissions, and growth specific factors in the context of Pakistan by using an annual time series data from 1970 to 2016 | Understanding | Nassani et al., 2018 |
| To analyze the nexus of water, energy and rice production system at the watershed scale. | Understanding | Ngammuangtueng et al., 2019 |
| Carried out quantitative and spatial assessments of FEW security in SSA using the Pardee Rand FEW Index. | Understanding | Nkiaka et al., 2021 |
| To analyze the factors affecting water-food-energy interconnection in Iran | Understanding | Norouzi 2022 |
| Explores the ecological indicators relevant to long-term sustainability by the food-energy, water nexus among BRICS (Brazil, the Russian Federation, India, China, and South Africa). | Understanding | Ozturk, 2015 |
| Examined the dynamic nexus between agricultural sustainability and food-energy-water poverty in a panel of selected SSA countries over the period of 1980–2013. | Understanding | Ozturk, 2017 |
| To evaluate the effect of long term CA with optimum nutrient management on energy budgeting, productivity, water and C-footprints, Water productivity (WP), and economics of the CA-based maize-wheat-mungbean system | Understanding | Parihar et al., 2022 |
| To analyze the interplay of water scarcity, relative energy abundance, and food production and consumption in the Kingdom of Saudi Arabia. | Understanding | Pieters and Swinnen, 2016 |
| To carry out an LCA study on various irrigation systems with a focus on pumping technologies independent of any crop-related issues. | Understanding | Pradeleix et al., 2015 |
| To simulate the effect of the trends of population change, economic growth and renewable energy sources development on water stress and groundwater security. | Understanding | Rahmani et al., 2022 |
| To assess the effect of capturing, treating and reusing wastewater for irrigation. | Understanding | Ramirez et al., 2021 |
| To assess and enhance the ecosystem provisioning services in Gavkhuni basin in central Iran. | Understanding | Ravar etal2020 |
| Evaluate a PES scheme from a multi-scalar and political-ecology perspective in order to reveal different power dynamics across the Water-Energy-Food (WEF) Nexus perspective. | Understanding | Rodríguez-de-Francisco et al., 2019 |
| Demonstrating successful performance in terms of determining and meeting water and energy requirements under constraints relative to aquifer sustainability | Understanding | Roje et al., 2020 |
| To examine how interdependent WEF infrastructural systems mediate the risks that climate extremes pose to urban WEF security. | Understanding | Romero-Lankao et al., 2018 |
| To investigate the oil palm biomass value chain in Peninsular Malaysia | Understanding | Rubinsin et al., 2021 |
| To raise knowledge of policy makers about the economic aspects of WFE systems in basin scale in countries in which detailed economic data of WFE systems is not available. | Understanding | Sharifinejad et al., 2020 |
| Applying causal structure-based framework at basin level, comparing the differences in historical and current causality of the WEFE nexus and water use | Understanding | Shi et al., 2021 |
| To analyse the major influencing factors, and water competition relationships in various sectors | Understanding | Shi et al., 2022 |
| To carry out multi-objective optimization, revealing the trade-off between the WEF benefits in the Upper Yellow River Basin | Understanding | Si et al., 2019 |
| To compare energy and carbon footprints of irrigation water provided by either a gravity-fed irrigation network requiring maintenance or a groundwater pumping system. | Understanding | Siyal et al., 2021 |
| To systematically and comprehensively analyze the influence mechanism of hydropower development in China according to an integrated analytical framework, quantitatively predicting the dynamic development pathways of hydropower under different scenarios | Understanding | Sun et al., 2021 |
| To quantitatively analyzing the tradeoffs and synergies of the WEF system in China | Understanding | Sun et al., 2022 |
| To explore the potential of MUSIASEM for resource nexus analysis at the river basin level. | Understanding | Taghdisian et al., 2022 |
| Examines the linkages between water resources and energy technologies at local level based on a case study conducted in Ouarzazate, Morocco, where one of the largest solar power complexes in the world was recently completed. | Understanding | Terrapon-Pfaff etal2021 |
| To characterize the watershed with respect to land use, climate, water resources, and other factors pertinent to the nexus and to explore the relationship between factors in the FEW nexus that may affect water management in terms of quality and availability | Understanding | Torres et al., 2020 |
| To study the outcomes of transboundary resource coordination | Understanding | Verhagen et al., 2021 |
| To quantitatively evaluate the sustainability of China's water-energy-food nexus | Understanding | Wang et al., 2018 |
| To identify how various districts in the Yangtze River contribute differently, and show interdependence upon, to the production and consumption of food, water, and energy. | Understanding | Wang et al., 2018 |
| Analyzes the spatial patterns and changing rules of different types of water system elements (including rivers/channels, reservoirs/lakes, ponds, and reed patches/tidal flats) both inside and surrounding the city. | Understanding | Wang et al., 2019 |
| To provide clustering maps based on the conventional density clustering approach and further integrates the evaluation of the inner building heterogeneity and the computational cost. S | Understanding | Wang et al., 2020 |
| To determine spatiotemporal differences and spatial convergence of the Water-Energy-Food-Ecology Nexus in Northwest China | Understanding | Wang et al., 2021 |
| To determine the main driving factors of water use and energy-related CO2 emissions at the regional scale from 1996 to 2016. | Understanding | Wangyong etal2021 |
| To analyze the interaction between land use (LU) types and water, energy, and food nexus attributes. | Understanding | Wolde et al., 2021 |
| To investigate the link between sustainable LWEF nexus and livelihoods. | Understanding | Wolde et al., 2022 |
| To assess the effects of climate change on agricultural production through the change in water availability, evaluating the adjustment responses and resulting energy consumption and GHG emission, with the Northeast China as a case study. | Understanding | Yan et al., 2018 |
| Evaluated the water, energy and food nexus (WEFN) in the Indus River of Pakistan | Understanding | Yang et al., 2016 |
| Identify and illustrate where development of new hydropower projects, upstream water diversions paths are in conflict | Understanding | Yang et al., 2016 |
| To reveal the hidden connections related to the food self-sufficiency issue, including the interdependencies of food production with its restraining factors (hybrid energy, hybrid water), other production sectors, and international exchanges. This | Understanding | Yiyi, 2019 |
| To give policy-makers insights into what efforts should be made towards sustainable agricultural management | Understanding | Yue and Guo, 2021 |
| To examine the non-linear relationship between water-energy food resources and air pollutants in a panel of 19 selected SSA countries, for a period of 2000–2014. | Understanding | Zaman et al., 2017 |
| To reflect the tradeoff between increscent demand due to economic development and limited supply capacity due to natural restriction and artificial localization. | Understanding | Zeng et al., 2019 |
| To evaluate the cross-sectional data static benefits of W-F input–output efficiency and W/F-E input–output efficiency in various regions of China from 2007 to 2016. | Understanding | Zhang and Xu, 2019 |
| To improve water-use efficiency from a WEF nexus perspective. | Understanding | Zhang et al., 2019 |
| The structural dynamics and complex relationships of the water-energy-food system in arid areas were analyzed from the perspective of the water footprint, and the risk characteristics were evaluated. | Understanding | Zhang et al., 2019 |
| To decompose the change of water use into factors, and analyze the contribution of each driver to identify the key drivers of total water use control. | Understanding | Zhang et al., 2021 |
| To address the following questions: (1) What are the Spatio-temporal dynamics of the expanded FEW nexus at the national and sub-national levels in China? (2) What are the two-way interactions of the expanded FEW nexus in China from the lens of sdgs? (3) What are the differences in the contribution of each sector to changes in other sectors in the expanded FEW nexus of China? | Understanding | Zhang et al., 2022 |
| To assess the agricultural production efficiency (APE) of seven provinces in the middle and lower reaches of the Yangtze River (MLYR) during 1996–2015. | Understanding | Zheng et al., , 2019 |
| To integrate existing water supply systems and small-hydropower installation | Understanding | Zhou et al., 2019 |
| To investigate the effects of marine resources on relieving the pressure of the food–energy–water (FEW) nexus in the coastal regions of China and its state changes | Understanding | Zhu et al., 2021 |
| To simulate the resource flow and social interactions of the complex water food energy system in the Ningdong Base | Understanding | Zhu et al., 2023 |
| [To analyze the interrelationships that exist between the water-energy-food nexus, the economic incentives and the preferences of those involved, as well as how they affect the entire system.](https://www.sciencedirect.com/topics/engineering/interrelationship) | Understanding | Villicaña-García et al., 2023 |
| To evaluate the coordinated development level of WEEF nexus in Tianjin, China. And the main obstacle factors that constrained the coordinated development of WEEF nexus | Understanding | Lv et al., 2023 |
| To evaluate the synergistic type, driver, and effect of water use for energy and food production in the Chinese mainland during 1997–2016 with multiple indicators. | Understanding | Hua et al., 2023 |
| Models the current state of the nexus in South Africa and explores future scenarios considering population growth, GDP per capita, and water and energy intensities. | Understanding | Nanfuka et al., 2023 |
| assesses water consumption, energy (EFs), and carbon footprints (CFs) associated with irrigation water supply for the major crops (wheat, rice, sugarcane, and cotton) | Understanding | Siyal et al., 2021 |
| To quantify and monitor the WEF nexus interlinkages at a national level. | Understanding | Simpson et al., 2022 |
| To quantify the benefits of proposed water resource development projects in the transboundary basin (4 storage and 7 run-of-the-river hydropower dams) in terms of hydroelectric power generation, crop production and flood damage reduction. | Understanding | Amjath-Babu et al., 2019 |
| To explore the FEW nexus in China’s Yangtze River Economic Belt. | Understanding | An et al., 2021 |
| Analysis of four cities with varying characteristics across the United States and India, to quantify system-wide water, energy/GHG, and land impacts associated with multiple food system actions to address health, equity, and environment. | Understanding | Boyer and Ramaswami, 2020 |
| To study the specific supply-and-demand mechanisms of water resources in each subsystem. | Understanding | Chen and Chen, 2020 |
| To show how such an integrated optimization, which takes maximum advantage of the spatial heterogeneity in resource abundance, could help resolve the conflicts around the water–food–energy (WFE) nexus and assist in its management. To quantify the production-possibility frontiers under different water-availability scenarios and demonstrate that in water-scarce regions, like Shanxi, the production capacity and corresponding production solutions are highly sensitive to water constraints. | Understanding | Gao et al., 2018 |
| To identify the relevant driving forces by the IOE-WEF in YREB, which is more consistent with the actual situation of YREB and better than the previous analyses. | Understanding | Ge et al., 2021 |
| To quantitatively assess water, energy and food security at the global scale in a holistic manner. | Understanding | Giupponi and Gain, 2017 |
| Exploring “Innovations in the Food-Energy-Water Nexus (INFEWS)” in the Columbia River Basin (CRB) through coordinated modeling and simulated management scenarios. | Understanding | Givens et al., |
| To model the global water system and explore the demand-availability dynamics, that is, the water stress, on a global and regional scale | Understanding | Hanumante et al., 2022 |
| To gain insight into interactions between all relevant sectors and ensure continuous development of adaptation or/and mitigation measures toward sustainable water resource management and human health. | Understanding | Hirwa et al., 2021 |
| To examine cooperative/competitive behaviors, including the water supply, water consumption, hydropower generation, and food crops growing in food-water-energy systems. | Understanding | Hu et al., 2018 |
| To investigate the coherence among the sectoral policies, either supporting or conflicting with each other, on cross-cutting strategies and their impacts on ecosystem and their services. | Understanding | Karabulut et al., 2019 |
| To analyze the GHG emissions from the current and future water supply scheme, as well as to draw a comparison between possible water reclamation with resource recovery scenarios in the town Leh in India. | Understanding | Lahmouri et al., 2019 |
| To examine effect of policy, innovation, governance, and labor as influencing factors and to understand how synergies, trade-offs, and long-overlooked interlinkages between sectors and among existing policies and institutions can become visible. | Understanding | Lazaro et al., 2021 |
| To evaluate the impact of Morocco’s phosphate industry on water, energy, and food sectors of Khouribga. | Understanding | Lee et al., 2020 |
| To study the harmonious balance of WEF and identify the main influencing factors, and further study the harmonious regulation of WEF | Understanding | Li et al., 2022 |
| To examine trade-offs described in policy, assessment, and operations documents to understand how health is framed in basin management regimes and where opportunities exist for proactively addressing health through management of FEW resources. | Understanding | Lund et al., 2022 |
| To investigate how farmers’ dynamic agricultural activities under different socio-economic conditions affect the WEF systems. | Understanding | Molajou et al., 2021 |
| To explore future climate scenarios on water, energy, food, and the environment in the Urmia Lake Basin using the WEFE nexus approach. | Understanding | Nasrollahi et al., 2021 |
| To predict production components, and a series of FEW index for achieving multi-objective solutions and quantitative assessments. | Understanding | Nie et al., 2018 |
| To evaluate the environmental performance of integrating a BNRT and a struvite recovery facility into the conventional sewage treatment system in Metro Manila. | Understanding | Pausta et al., 2019 |
| Measure the comprehensive evaluation index and coupling coordination degree of China’s 30 provinces from 2005 to 2017. | Understanding | Qi et al., 2022 |
| To identify key points of WEF nexus management in the BRI from WEF utilization efficiency, final demand behavior and trade inequality. | Understanding | Qian et al., 2022 |
| To increase understanding of the interlinkages in the water, energy, and food nexus, explains why it is important to consider this nexus in the context of adaptation responses, and argues that focusing on trade-offs and synergies using a nexus approach could facilitate greater climate change adaptation and help ensure food, water, and energy security by enhancing resource use efficiency and encouraging greater policy coherence. | Understanding | Rasul and Sharma, 2016 |
| Conduct a detailed life cycle analysis for solar PV, aloe vera gel production, and a hypothetical colocated solar–aloe system to explore the tradeoffs and synergies (in the context of energy, water, and greenhouse gas emissions) between these two emerging land uses in northwestern India. To examine the economics of these systems and the potential for improving rural livelihoods (employment generation, rural electrification). | Understanding | Ravi et al., 2016 |
| To explore the potential effects of electricity pricing on crop cultivation in Saudi Arabia. | Understanding | Shannak et al., 2022 |
| To quantify the cross sectoral interactions between water, energy, and food in Vietnam | Understanding | Shivakumar et al., 2021 |
| To investigate the water resources in the Upper Blue Nile River (UBNR) basin, one of the two main sources of the Nile | Understanding | Stamou and Rutschmann, 2018 |
| Analyse how actors involved in the governance of water, energy and food systems are embedded in social networks, and discuss how that embeddedness shapes collaboration and coordination processes that are relevant for addressing interconnected sustainability challenges. | Understanding | Stein et al., 2018 |
| Apply innovative assessment methods to analyze the sustainability of WEF system and the coupling coordinated level of WEF nexus in different regions of China. | Understanding | Sun et al., 2022 |
| To understand a few aspects of the complex relationship among climate–ecosystem–human systems within the integrated modeling framework. | Understanding | Tian et al., 2018 |
| Explore the interaction between subsystems in WEFE system under green development based on pressure-state-response (PSR) model; to assess the coupling coordination development level of WEFE system in the YREB from 2008 to 2019 on the basis of the construction of evaluation index system. Apply the obstacle degree model to analyze the main constraints that affected the coordination development of regional WEFE system. | Understanding | Tonghui and Junfei, 2022 |
| To clarify the nexus between WEF resources and its impact on the development of the entire region. | Understanding | Wang et al., 2022 |
| To characterize and visualize EWL resource use, EWL system linkages within and among sub-regions, and the EWL nexus implications of future policies and investments. | Understanding | Wild etal2021 |
| Identify WEF nexus resource driving factor indicators that affect nexus resources, (ii) explore the extent and trends of a driver on four nexus resources, and (iii) identify the direct and indirect impact of driving factors on LWEF nexus resources | Understanding | Wolde et al., 2021 |
| To quantifying natural and human components that affect the WEF nexus in the Great Ruaha River system. | Understanding | Yang and Wi, 2018 |
| To explore the tele-coupling mechanism of urban FEW nexus that could help to promote coordinating resource management for city and its hinterlands. | Understanding | Zhang et al., 2019 |
