# Supplementary figures and images for: Review of water–energy–food nexus applications in the Global South
Source: Camb Prism Water. Author manuscript; Available in PMC 2025 Apr 3. (PMC7617535; doi:10.1017/wat.2024.8)

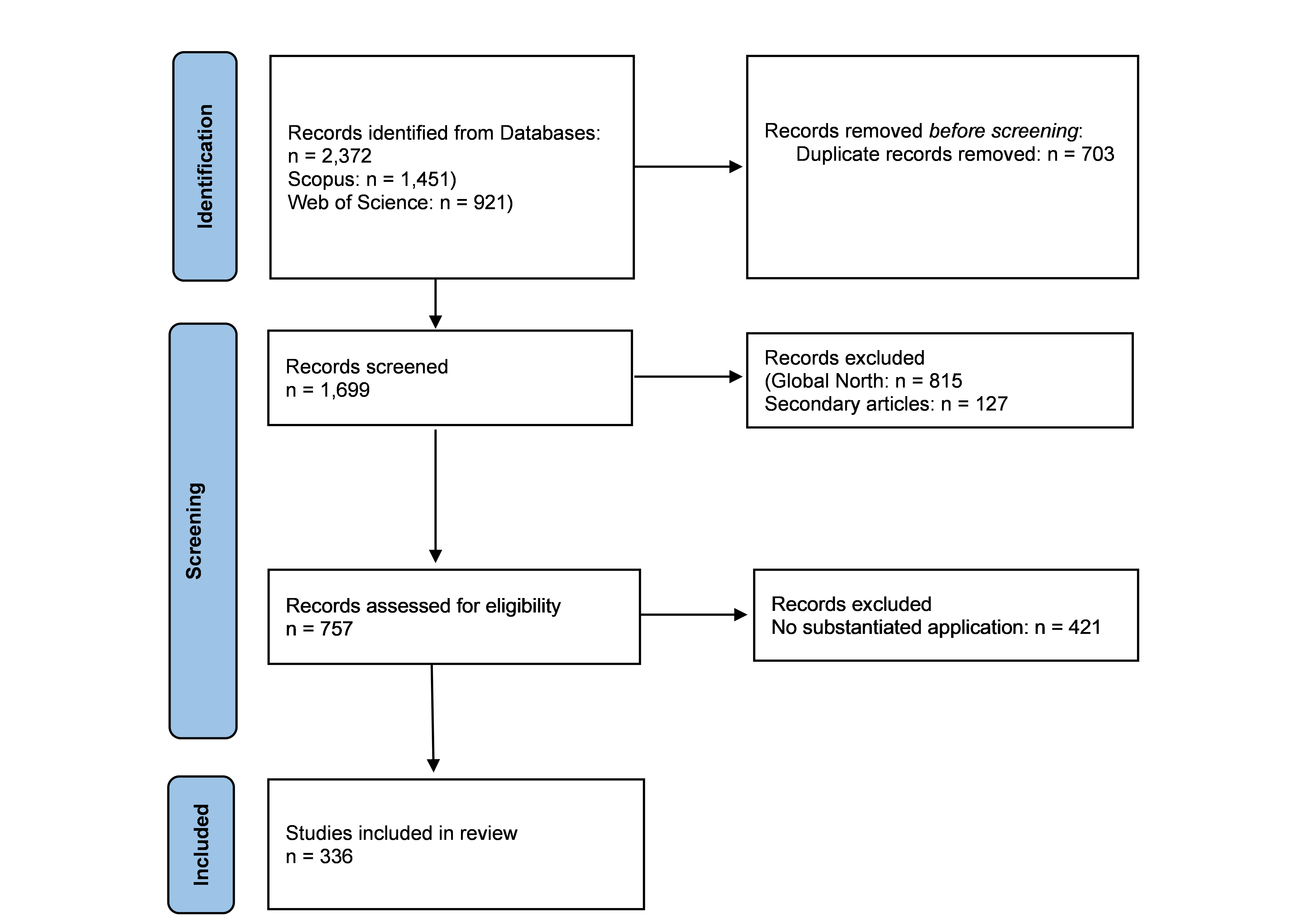

Supplement: supplementary material 2 [file EMS203570-supplement-supplementary_material_2.tif]
